# Supplementary material for: Comparison between Effects of Retroactivity and Resource Competition upon Change in Downstream Reporter Genes of Synthetic Genetic Circuits
Source: Life (Basel). 2019 Mar 26;9(1):30. doi: 10.3390/life9010030 (PMC6463139; doi:10.3390/life9010030)
Supplement: Supplementary file 1 [file life-09-00030-s001.zip › 0320 supplymentary/supporting_information.pdf]

Comparison between effects of retroactivity and resource competition upon change in downstream reporter genes of synthetic genetic circuits

Supporting Information

Takefumi Moriya[1], Tomohiro Yamaoka[2], Yuki Wakayama[2], Shotaro Ayukawa[3], Zicong Zhang[1], Masayuki Yamamura[1], Shinji Wakao[2], and Daisuke Kiga[1,2,*]

[1] Department of Computational Intelligence and Systems Science, Tokyo Institute of Technology, Yokohama, Kanagawa 226-8503, Japan.

[2] Department of Electrical Engineering and Bioscience, Waseda University, Shinjuku, Tokyo, 169-8050, Japan.

[3] Waseda Research Institute for Science and Engineering, Waseda University, Shinjuku, Tokyo, 169-8050, Japan.

* Correspondence: kiga@waseda.jp

1. Supplementary Methods

Supplementary Methods 1. Detailed mathematical modeling of chemical reactions and simulation.
Supplementary Methods 2. Detailed imaging process.

2. Supplementary Figure S1-12

Figure S1. Transition of Protein-binding status in promoters and decoy sites.
Figure S2. DNA sequences of the plasmids and primers.
Figure S3. Scheme of imaging process.
Figure S4. Composition of lac promoter.
Figure S5. Fluorescence intensity in the constitutive GFP expressed condition.
Figure S6. Cumulative relative frequency distributions of the relative bottom counts for the constitutive and oscillation condition.
Figure S7. Cumulative relative frequency distributions of bottom counts for the lac/ara-reporter circuit strain.
Figure S8. Cumulative relative frequency distributions of bottom counts for the lac -reporter circuit strain.
Figure S9. Oscillation periods in the presence or absence of downstream molecular competitions by deterministic simulation.
Figure S10. Oscillation Amplitude of total proteins (AraC, LacI, and GFP) in the presence or absence of downstream molecular competitions by deterministic simulation.
Figure S11. Amplitude of AraC dimer molecule oscillations in the presence or absence of downstream molecular competitions by deterministic simulation.
Figure S12. Amplitude of GFP monomer molecule oscillations in the presence or absence of downstream molecular competitions by deterministic simulation.

3. Supplementary Movie SM1-5

Movie SM1. Microscopy image stack movie of the lac/ara-reporter circuit strain with 1.0% arabinose and 0.01 mM IPTG.
Movie SM2. Microscopy image stack movie of the lac-reporter circuit strain with 1.0% arabinose and 0.01 mM IPTG.
Movie SM3. Microscopy image stack movie of the lac/ara-reporter circuit strain with 1.0% arabinose and 10 mM IPTG.
Movie SM4. Microscopy image stack movie of the lac-reporter circuit strain with 1.0% arabinose and 10 mM IPTG.
Movie SM5. Microscopy image stack movie of the GFP constitutive expression (Ptet-*gfp* strain).

1. Supplementary Methods

Supplementary Methods 1 - Detailed mathematical modeling of chemical reactions and simulation.

The dynamics of interaction with protein-binding sites in the promoters were determined according to the following set of reactions for $P^{i}_{j,k,l}$ (Figure 3): P represents the promoter, i accounts for the coding sequence for the protein expression promoter of the activator (a: AraC), repressor (r: LacI) and downstream (d: GFP) promoters. In addition, j and k represent the number of proteins bound to the promoter. Also, $j \in \{0, 1\}$ accounts for the binding of AraC dimers (a_2) and $k \in \{0, 1, 2\}$ represents binding of LacI tetramers (r_4). Furthermore, $l \in \{0, L\}$ represents the looping status for the promoter: $P^{i}_{j,k,0}$ in the unlooped state of the promoters and $P^{i}_{j,k,L}$ is the looped state of the promoters. The dynamics of interaction with protein-binding sites in the decoy sites were determined according to the following set of reactions for D_j: D represents the AraC-binding decoy sites, j represents the number of proteins bound to the decoy sites and $j \in \{0, 1\}$ accounts for the binding of AraC dimers (a_2). All the parameters used in this study are listed in Table A1.

Protein binding to DNA was calculated according to the formulas described below. In all three models (lac/ara-reporter circuit, lac-reporter circuit and lac-reporter + AraC decoy circuit models), the binding rate of LacI tetramers to DNA and the dissociation rate from DNA was

described as k_r and k_{-r}. Similarly, the binding rate of AraC dimers to DNA and the dissociation rate from DNA are described as k_a and k_{-a}. IPTG binds to the LacI repressor and reduces its affinity for the protein-binding sites. Arabinose binds to the AraC activator and increases its affinity for the protein-binding sites. Using the maximum affinity of regulatory proteins for these protein-binding sites (C_r^{max} = 0.2 molecules^{-1} and C_a^{max} = 1 molecules^{-1}) and the minimum affinity for the promoters (C_r^{min} = 0.01 molecules^{-1} and C_a^{min} = 0 molecules^{-1}), k_r and k_a can be calculated from the following equations:

$$k_r = k_{-r}\left(\left(C_r^{max} - C_r^{min}\right)\frac{1}{1+\left(\frac{[IPTG]}{k_{r1}}\right)^{b_1}} + C_r^{min}\right) \quad (1)$$

$$k_a = \mathrm{k_{-a}}\left(\left(C_a^{max} - C_a^{min}\right)\frac{[ara]^{c_1}}{k_{a1}^{c_1}+[ara]^{c_1}}\frac{1}{1+\left(\frac{[IPTG]}{k_{r2}}\right)^{b_2}} + C_a^{min}\right) \quad (2)$$

In the AraC-coding lac/ara promoter and LacI-coding lac/ara promoter in all three circuits (Figure 2), binding of the AraC dimer to DNA and dissociation from DNA, binding of the LacI tetramer to DNA and dissociation from DNA, as well as DNA looping/unlooping, are described as follows:

$$P_{j,1,0}^i \underset{k_{-l}}{\overset{k_l}{\rightleftarrows}} P_{j,1,L}^i \qquad i \in \{a,r\}, j \in \{0,1\} \quad (3)$$

$$P_{j,0,L}^i \xrightarrow{k_{ul}} P_{j,0,0}^i \qquad i \in \{a,r\}, j \in \{0,1\} \quad (4)$$

$$P_{j,0,0}^i + r_4 \underset{k_{-r}}{\overset{2k_r}{\rightleftarrows}} P_{j,1,0}^i \qquad i \in \{a,r\}, j \in \{0,1\} \quad (5)$$

$$P_{j,1,0}^i + r_4 \underset{2k_{-r}}{\overset{k_r}{\rightleftarrows}} P_{j,2,0}^i \qquad i \in \{a,r\}, j \in \{0,1\} \quad (6)$$

$$P_{0,k,0}^i + a_2 \underset{k_{-a}}{\overset{k_a}{\rightleftarrows}} P_{1,k,0}^i \qquad i \in \{a,r\}, k \in \{0,1,2\} \quad (7)$$

$$P^{i}_{0,k,L} + a_2 \underset{k_{-a}}{\overset{k_a}{\rightleftarrows}} P^{i}_{1,k,L} \qquad i \in \{a,r\}, k \in \{0,1\} \tag{8}$$

In the GFP-coding lac/ara promoter in the lac/ara-reporter circuit, binding of the AraC dimer to DNA and dissociation from DNA, binding of the LacI tetramer to DNA and dissociation from DNA, as well as DNA looping/unlooping, are described as follows:

$$P^{d}_{j,1,0} \underset{k_{-l}}{\overset{k_l}{\rightleftarrows}} P^{d}_{j,1,L} \qquad j \in \{0,1\} \tag{9}$$

$$P^{d}_{j,0,L} \overset{k_{ul}}{\rightarrow} P^{d}_{j,0,0} \qquad j \in \{0,1\} \tag{10}$$

$$P^{d}_{j,0,0} + r_4 \underset{k_{-r}}{\overset{2k_r}{\rightleftarrows}} P^{d}_{j,1,0} \qquad j \in \{0,1\} \tag{11}$$

$$P^{d}_{j,1,0} + r_4 \underset{2k_{-r}}{\overset{k_r}{\rightleftarrows}} P^{d}_{j,2,0} \qquad j \in \{0,1\} \tag{12}$$

$$P^{d}_{0,k,0} + a_2 \underset{k_{-a}}{\overset{k_a}{\rightleftarrows}} P^{d}_{1,k,0} \qquad k \in \{0,1,2\} \tag{13}$$

$$P^{d}_{0,k,L} + a_2 \underset{k_{-a}}{\overset{k_a}{\rightleftarrows}} P^{d}_{1,k,L} \qquad k \in \{0,1\} \tag{14}$$

In the GFP-coding lac promoter in the lac-reporter and lac-reporter + AraC decoy circuits, binding of the LacI tetramer to DNA and dissociation from DNA, as well as DNA looping/unlooping, are described as follows:

$$P^{d}_{0,1,0} \underset{k_{-l}}{\overset{k_l}{\rightleftarrows}} P^{d}_{0,1,L} \tag{15}$$

$$P^{d}_{0,0,L} \overset{k_{ul}}{\rightarrow} P^{d}_{0,0,0} \tag{16}$$

$$P^{d}_{0,0,0} + r_4 \underset{k_{-r}}{\overset{2k_r}{\rightleftarrows}} P^{d}_{0,1,0} \tag{17}$$

$$P^{d}_{0,1,0} + r_4 \underset{2k_{-r}}{\overset{k_r}{\rightleftarrows}} P^{d}_{0,2,0} \tag{18}$$

In the additional AraC decoy sites in the lac-reporter + AraC decoy circuits, binding of the AraC

dimer to DNA and dissociation from DNA are described as follows:

$$D_0 + a_2 \underset{k_{-a}}{\overset{k_a}{\rightleftarrows}} D_1 \quad (19)$$

In the absence of downstream competition for regulatory proteins (Figures 8D–F, J–L, S8D–F, J–L, S9D–F, J–L, S10D–F and J–L), we defined the number of a_2 (AraC dimer) and r_4 (LacI tetramer) molecules as unchanged in either forward or backward reactions (9)–(19) of protein bindings for GFP production and AraC-binding to the decoy. To describe those 'bindings' do not change the number of free AraC and LacI molecules.

The dynamics of mRNA transcription were determined according to the following set of reactions:

For all three circuits,

$$P^a_{0,0,0} \overset{b_a}{\rightarrow} P^a_{0,0,0} + m^a \quad (20)$$

$$P^r_{0,0,0} \overset{b_r}{\rightarrow} P^r_{0,0,0} + m^r \quad (21)$$

$$P^a_{1,0,0} \overset{\alpha b_a}{\rightarrow} P^a_{1,0,0} + m^a \quad (22)$$

$$P^r_{1,0,0} \overset{\alpha b_r}{\rightarrow} P^r_{1,0,0} + m^r \quad (23)$$

For the lac/ara-reporter circuit,

$$P^d_{0,0,0} \overset{b_{d_1}}{\rightarrow} P^d_{0,0,0} + m^d \quad (24)$$

$$P^d_{1,0,0} \overset{\alpha b_{d_1}}{\rightarrow} P^d_{1,0,0} + m^d \quad (25)$$

For the lac-reporter and lac-reporter + AraC decoy circuits,

$$P^d_{0,0,0} \overset{b_{d_2}}{\rightarrow} P^d_{0,0,0} + m^d \tag{26}$$

where m^i represents the number of activators/repressors/downstream mRNA and i accounts for the coding sequence for the protein expression promoter of the activator (a: AraC), repressor (r: LacI) and downstream (d: GFP).

The dynamics of protein synthesis were determined according to the following set of reactions:

$$m^a \overset{t_a}{\rightarrow} m^a + a_{uf} \tag{27}$$

$$m^r \overset{t_r}{\rightarrow} m^r + r_{uf} \tag{28}$$

$$m^d \overset{t_d}{\rightarrow} m^d + d_{uf} \tag{29}$$

$$a_{uf} \overset{k_{fa}}{\rightarrow} a \tag{30}$$

$$r_{uf} \overset{k_{fr}}{\rightarrow} r \tag{31}$$

$$d_{uf} \overset{k_{fd}}{\rightarrow} d \tag{32}$$

$$a + a \underset{k_{-da}}{\overset{k_{da}}{\rightleftarrows}} a_2 \tag{33}$$

$$r + r \underset{k_{-dr}}{\overset{k_{dr}}{\rightleftarrows}} r_2 \tag{34}$$

$$r_2 + r_2 \underset{k_{-t}}{\overset{k_t}{\rightleftarrows}} r_4 \tag{35}$$

where m^i represents the number of activators/repressors/downstream mRNA, i accounts for the coding sequence for the protein expression promoter of the activator (a: AraC), repressor (r: LacI) and downstream (d: GFP) and X_{uf} represent the number of activators/repressors/downstream

unfolded polypeptides, respectively. a , r and d represent the number of activators/repressors/downstream folded monomeric proteins, respectively; a_2 and r_2 represent the number of activators/repressors for folded dimeric proteins, respectively; and r_4 represents the number of repressors for folded tetrameric proteins.

The dynamics of decay of the components were controlled by the sum of the target proteins.

In all circuits,

$$f(X) = \frac{\gamma}{c_e + \omega_a X_a + \omega_r X_r + \omega_d X_d} \quad (36)$$

For the lac/ara-reporter circuit,

$$X_a = a_{uf} + a + 2a_2 + 2\sum_{i=1}^{3}\sum_{k=0}^{2} P_{1,k,0}^{i} + 2\sum_{i=1}^{3}\sum_{k=0}^{1} P_{1,k,L}^{i}$$

$$i \in \{a, r, d\}, k \in \{0, 1, 2\} \quad (37)$$

$$X_r = r_{uf} + r + 2r_2 + 4r_4 + 4\sum_{i=1}^{3}\sum_{j=0}^{1}\sum_{l=0}^{1} P_{j,1,l}^{i} + 8\sum_{i=1}^{3}\sum_{j=0}^{1} P_{j,2,0}^{i}$$

$$i \in \{a, r, d\}, j \in \{0,1\}, l \in \{0, L\} \quad (38)$$

$$X_d = d_{uf} + d \quad (39)$$

For the lac-reporter circuit,

$$X_a = a_{uf} + a + 2a_2 + 2\sum_{i=1}^{3}\sum_{k=0}^{2} P_{1,k,0}^{i} + 2\sum_{i=1}^{3}\sum_{k=0}^{1} P_{1,k,L}^{i}$$

$$i \in \{a, r, d\}, k \in \{0, 1, 2\} \quad (40)$$

$$X_r = r_{uf} + r + 2r_2 + 4r_4 + 4\sum_{i=1}^{3}\sum_{j=0}^{1}\sum_{l=0}^{1} P_{j,1,l}^{i} + 8\sum_{i=1}^{3}\sum_{j=0}^{1} P_{j,2,0}^{i}$$

$$i \in \{a, r, d\}, j \in \{0,1\}, l \in \{0, L\} \quad (41)$$

$$X_d = d_{uf} + d \qquad l \in \{0, L\} \quad (42)$$

For the lac-reporter + AraC decoy circuit,

$$X_a = a_{uf} + a + 2a_2 + 2D_1 + 2\sum_{i=1}^{3}\sum_{k=0}^{2} P_{1,k,0}^{i} + 2\sum_{i=1}^{3}\sum_{k=0}^{1} P_{1,k,L}^{i}$$

$$i \in \{a, r, d\}, k \in \{0, 1, 2\} \quad (43)$$

$$X_r = r_{uf} + r + 2r_2 + 4r_4 + 4\sum_{i=1}^{3}\sum_{j=0}^{1}\sum_{l=0}^{1} P_{j,1,l}^{i} + 8\sum_{i=1}^{3}\sum_{j=0}^{1} P_{j,2,0}^{i}$$

$$i \in \{a, r, d\}, j \in \{0,1\}, l \in \{0, L\} \quad (44)$$

$$X_d = d_{uf} + d \quad (45)$$

In the absence of downstream competition for proteases (Figures 5G–L, S4G–L, S5G–L and S6G–L), we set ω_d as zero to describe that degradation of GFP does not affect AraC and LacI.

The dynamics of decay of the components were described in the following reactions:

In all circuits,

$$m^a \xrightarrow{d_a} \emptyset \quad (46)$$

$$m^r \xrightarrow{d_r} \emptyset \quad (47)$$

$$m^d \xrightarrow{d_d} \emptyset \quad (48)$$

$$a_{uf} \xrightarrow{\lambda f(X)} \emptyset \quad (49)$$

$$r_{uf} \xrightarrow{f(X)} \emptyset \quad (50)$$

$$d_{uf} \xrightarrow{\lambda f(X)} \emptyset \quad (51)$$

$$a \xrightarrow{\lambda f(X)} \emptyset \quad (52)$$

$$r \xrightarrow{f(X)} \emptyset \quad (53)$$

$$d \xrightarrow{\lambda f(X)} \emptyset \quad (54)$$

$$a_2 \xrightarrow{\lambda f(X)} \emptyset \quad (55)$$

$$r_2 \xrightarrow{f(X)} \emptyset \quad (56)$$

$$r_4 \xrightarrow{f(X)} \emptyset \quad (57)$$

For the AraC- and LacI-coding lac/ara promoters in all circuits,

$$P^i_{j,1,0} \xrightarrow{f(X)} P^i_{j,0,0} \quad i \in \{a, r\}, j \in \{0, 1\} \quad (58)$$

$$P^i_{j,1,L} \xrightarrow{\varepsilon f(X)} P^i_{j,0,L} \quad i \in \{a, r\}, j \in \{0, 1\} \quad (59)$$

$$P^i_{j,2,0} \xrightarrow{2 f(X)} P^i_{j,1,0} \quad i \in \{a, r\}, j \in \{0, 1\} \quad (60)$$

$$P^i_{1,k,0} \xrightarrow{\lambda f(X)} P^i_{0,k,0} \quad i \in \{a, r\}, k \in \{0, 1, 2\} \quad (61)$$

$$P^i_{1,k,L} \xrightarrow{\lambda f(X)} P^i_{0,k,L} \quad i \in \{a, r\}, k \in \{0, 1\} \quad (62)$$

For the GFP-coding promoter in the lac/ara-reporter circuit,

$$P^d_{j,1,0} \xrightarrow{f(X)} P^d_{j,0,0} \quad j \in \{0, 1\} \quad (63)$$

$$P^d_{j,1,L} \xrightarrow{\varepsilon f(X)} P^d_{j,0,L} \quad j \in \{0, 1\} \quad (64)$$

$$P^d_{j,2,0} \xrightarrow{2 f(X)} P^d_{j,1,0} \quad j \in \{0, 1\} \quad (65)$$

$$P^d_{1,k,0} \xrightarrow{\lambda f(X)} P^d_{0,k,0} \quad k \in \{0, 1, 2\} \quad (66)$$

$$P^d_{1,k,L} \xrightarrow{\lambda f(X)} P^d_{0,k,L} \quad k \in \{0, 1\} \quad (67)$$

For the GFP-coding promoter in the lac-reporter circuit and lac-reporter + AraC decoy circuit,

$$P^{d}_{0,1,0} \quad \overset{f(X)}{\rightarrow} \quad P^{d}_{0,0,0} \tag{68}$$

$$P^{d}_{0,1,L} \quad \overset{\varepsilon f(X)}{\rightarrow} \quad P^{d}_{0,0,L} \tag{69}$$

$$P^{d}_{0,2,0} \quad \overset{2f(X)}{\rightarrow} \quad P^{d}_{0,1,0} \tag{70}$$

For the additional AraC decoy sites in the lac-reporter + AraC decoy circuit,

$$D_1 \quad \overset{\lambda f(X)}{\rightarrow} \quad D_0 \tag{71}$$

Copy number variations ($N_i,\ i = a,\ r,\ d, decoy$) were accounted for as follows:

For the AraC- and LacI-coding promoters in all circuits,

$$P^{a}_{0,0,L} = N_a - (P^{a}_{0,0,0} + P^{a}_{0,1,0} + P^{a}_{0,2,0} + P^{a}_{1,0,0} + P^{a}_{1,1,0} + P^{a}_{1,2,0} + P^{a}_{0,1,L} + P^{a}_{1,0,L} + P^{a}_{1,1,L}) \tag{72}$$

$$P^{r}_{0,0,L} = N_r - (P^{r}_{0,0,0} + P^{r}_{0,1,0} + P^{r}_{0,2,0} + P^{r}_{1,0,0} + P^{r}_{1,1,0} + P^{r}_{1,2,0} + P^{r}_{0,1,L} + P^{r}_{1,0,L} + P^{r}_{1,1,L}) \tag{73}$$

For the GFP-coding promoter in the lac/ara-reporter circuit,

$$P^{d}_{0,0,L} = N_d - (P^{d}_{0,0,0} + P^{d}_{0,1,0} + P^{d}_{0,2,0} + P^{d}_{1,0,0} + P^{d}_{1,1,0} + P^{d}_{1,2,0} + P^{d}_{0,1,L} + P^{d}_{1,0,L} + P^{d}_{1,1,L}) \tag{74}$$

For the GFP-coding promoter in the lac-reporter and lac-reporter + AraC decoy circuits,

$$P^{d}_{0,0,L} = N_d - (P^{d}_{0,0,0} + P^{d}_{0,1,0} + P^{d}_{0,2,0} + P^{d}_{0,1,L}) \tag{75}$$

For copy number variations of AraC decoy sites in the lac-reporter + AraC decoy circuit,

$D_0 = N_{decoy} - D_1$ (76)

The deterministic model was simulated using MATLAB software (MathWorks). Our ODE simulations were performed using ode45 solver in MATLAB. The initial values in the lac/ara-reporter circuit model were configured as [$P^a_{0,0,0}$, $P^a_{0,1,0}$, $P^a_{0,2,0}$, $P^a_{1,0,0}$, $P^a_{1,1,0}$, $P^a_{1,2,0}$, $P^a_{0,1,L}$, $P^a_{1,0,L}$, $P^a_{1,1,L}$, $P^r_{0,0,0}$, $P^r_{0,1,0}$, $P^r_{0,2,0}$, $P^r_{1,0,0}$, $P^r_{1,1,0}$, $P^r_{1,2,0}$, $P^r_{0,1,L}$, $P^r_{1,0,L}$, $P^r_{1,1,L}$, $P^d_{0,0,0}$, $P^d_{0,1,0}$, $P^d_{0,2,0}$, $P^d_{1,0,0}$, $P^d_{1,1,0}$, $P^d_{1,2,0}$, $P^d_{0,1,L}$, $P^d_{1,0,L}$, $P^d_{1,1,L}$, m^a, m^r, m^d, a_{uf}, r_{uf}, d_{uf}, a, r, d, a_2, r_2, r_4 = 0, 0, 0, 0, 0, 0, 0, 0, 0, 0, 0, 0, 0, 0, 0, 0, 0, 0, 0, 0, 0, 0, 0, 0, 0, 0, 0, 0, 0, 0, 0, 0, 0, 0, 0, 0, 0, 0, 0 (molecules)]. The initial values in the lac-reporter circuit model were configured as [$P^a_{0,0,0}$, $P^a_{0,1,0}$, $P^a_{0,2,0}$, $P^a_{1,0,0}$, $P^a_{1,1,0}$, $P^a_{1,2,0}$, $P^a_{0,1,L}$, $P^a_{1,0,L}$, $P^a_{1,1,L}$, $P^r_{0,0,0}$, $P^r_{0,1,0}$, $P^r_{0,2,0}$, $P^r_{1,0,0}$, $P^r_{1,1,0}$, $P^r_{1,2,0}$, $P^r_{0,1,L}$, $P^r_{1,0,L}$, $P^r_{1,1,L}$, $P^d_{0,0,0}$, $P^d_{0,1,0}$, $P^d_{0,2,0}$, $P^d_{0,1,L}$, m^a, m^r, m^d, a_{uf}, r_{uf}, d_{uf}, a, r, d, a_2, r_2, r_4 = 0, 0, 0, 0, 0, 0, 0, 0, 0, 0, 0, 0, 0, 0, 0, 0, 0, 0, 0, 0, 0, 0, 0, 0, 0, 0, 0, 0, 0, 0, 0, 0, 0, 0 (molecules)]. The initial values in the lac-reporter + AraC decoy circuit model were configured as [$P^a_{0,0,0}$, $P^a_{0,1,0}$, $P^a_{0,2,0}$, $P^a_{1,0,0}$, $P^a_{1,1,0}$, $P^a_{1,2,0}$, $P^a_{0,1,L}$, $P^a_{1,0,L}$, $P^a_{1,1,L}$, $P^r_{0,0,0}$, $P^r_{0,1,0}$, $P^r_{0,2,0}$, $P^r_{1,0,0}$, $P^r_{1,1,0}$, $P^r_{1,2,0}$, $P^r_{0,1,L}$, $P^r_{1,0,L}$, $P^r_{1,1,L}$, $P^d_{0,0,0}$, $P^d_{0,1,0}$, $P^d_{0,2,0}$, $P^d_{0,1,L}$, m^a, m^r, m^d, a_{uf}, r_{uf}, d_{uf}, a, r, d, a_2, r_2, r_4, D_1 = 0, 0, 0, 0, 0, 0, 0, 0, 0, 0, 0, 0, 0, 0, 0, 0, 0, 0, 0, 0, 0, 0, 0, 0, 0, 0, 0, 0, 0, 0, 0, 0, 0, 0, 0 (molecules)].

Supplementary Methods 2 - Detailed imaging process.

For the analysis of oscillation dynamics, we performed microcolony recognition,

background subtraction and fluorescence quantification (Figure S2). These steps allowed us to plot heat maps of the oscillation damping of 310 microcolonies. Each microcolony comprised hundreds of cells at the last time point. We used DIC and fluorescence images, which revealed 1–3 microcolonies each.

Microcolony recognition was performed first using three filtering steps and one binary processing step. Background subtraction between the recognised microcolony and its neighbouring area was used to reduce irregular excitation and photon noise, which varies with Poisson distribution in the background throughout an image.

In the first process of microcolony recognition, each DIC image (512 × 512 pixel, 16-bit) was sharpened with a weighted average of the 3 × 3 neighbourhood [−1 −1 −1; −1 +12 −1; −1 −1 −1]. The second process of microcolony recognition was convolution by the kernel [−1 −1 −1 −1 −1; −1 −1 −1 −1 −1; −1 −1 24 −1 −1; −1 −1 −1 −1 −1; −1 −1 −1 −1 −1]. In the third process, a Gaussian blur was performed with a radius of 4.0. Finally, Otsu banalization was performed to obtain the shapes of microcolonies in DIC images. If binarised microcolonies collided with each other at any time point, the microcolonies were excluded from the following analysis. Binarised colonies at the last time point have approximately 1,000–100,000 pixels in an area.

For background subtraction, the background fluorescence recognised by the binarised shapes of microcolonies was subtracted from raw data. To fix the background fluorescence intensity

for each pixel of a microcolony, we defined the 'doughnut' area regarding the subtracted shape of the microcolony from the 20 pixel-enlarged shapes in the binarised DIC image. The fluorescence intensity of the microcolony was then substituted by the average fluorescent intensity of the neighbouring 'doughnut' areas. Next, the entire fluorescent image was applied to four median filters (50, 50, 50 and 10 pixels), to obtain the background fluorescence at each pixel for each microcolony. This background fluorescence was subtracted from the raw data to obtain fluorescence intensities from cells.

The first process of fluorescence quantification was a time course fluorescent measurement of the cell trajectories in microcolonies (Figure S2-1). We obtained 15,768 lineages among 273 microcolonies using the microcolony-growth tracking step and trajectory determination step using the information of microcolony-growth. In the microcolony-growth tracking step, the same colony between time points was recognised by manual backtracking with the ImageJ plugin MTrackJ. Note that one image can contain multiple microcolonies. In the following trajectory determination step, the growth of the microcolony and change of its centroid were key factors to find trajectories of cell lineages. To obtain the start position for backtracking of a cell lineage, we randomly allocated an XY coordinate and angle of a rectangle with a 3 × 2 pixel cell, corresponding to the size of *E. coli*, on the image for the final time point of the culture. If one or more pixels of the rectangle were outside the microcolonies, we cancelled the allocation and randomly allocated

another rectangle. For such rectangles inside a microcolony, the distance vector was defined between the centroid positions of the microcolony and the rectangle.

$$\overrightarrow{x_{t=n}} = \overrightarrow{c_{t=n}} + \overrightarrow{L_{t=n}}$$

(x: centroid of a rectangle, c: centroid of a microcolony, L: distance vector, n: frame number)

The backtrack algorithm used the following rules, including information for microcolony growth between the current and past frames:

$$\overrightarrow{x_{t=n-1}} = \overrightarrow{c_{t=n-1}} + \overrightarrow{L_{t=n-1}} = \overrightarrow{c_{t=n-1}} + \overrightarrow{L_{t=n}}\frac{S_n}{S_{n-1}} = \overrightarrow{c_{t=n-1}} + (\overrightarrow{x_{t=n}} - \overrightarrow{c_{t=n}})\frac{S_n}{S_{n-1}}$$

(S: area of a microcolony)

If a backtracked position was not in the same microcolony in the time series, the backtracked position was abandoned and another random allocation of a rectangle on the final time point image was attempted. For the total time series of images of all inducer conditions of two strains, we tried 961,848 allocations to obtain 15,768 lineages in this study. We measured the fluorescence intensity of each trajectory by subtracting the background from the time-course fluorescence images.

The second process of fluorescence quantification was a bottom count determination based on the fluorescence time course of each lineage in an inducer condition of a strain (Figure S2-2). To distinguish between noise and oscillation, a 12 per min moving average was determined from the 180 min fluorescent intensity time series for a lineage. We counted oscillation bottom points of the moving average time course. Each temporal oscillation bottom point was defined as a point with

smaller fluorescence intensity than those of both of the points just before and after. Each temporal oscillation peak point was similarly defined. We excluded temporal bottom points with higher fluorescence intensity than the 0.2 fold of the fluorescence intensity of either neighbouring temporal oscillation peak point (local maxima).

The third process of fluorescence quantification was plotting the bottom counts of the cumulative relative frequency distribution for one inducer condition of one strain (Figure S2-3). Bottom counts were sorted in descending order and plotted in the cumulative relative frequency distribution. The ratio of the three or more bottom point counts was calculated from this distribution in the inducer condition of a strain.

The final process of fluorescence quantification was plotting of a colour map of the ratio of the three or more bottom counts in all of the inducer conditions of two strains (Figure S2-4). For all inducer conditions (arabinose: 0.01%, 0.1% or 1.0%; IPTG: 0, 0.01, 0.1, 1.0 or 10 mM), we plotted calculations of the relative frequency using the pseudocolour 'jet' from MATLABs built-in colour.

2. Supplementary Figures

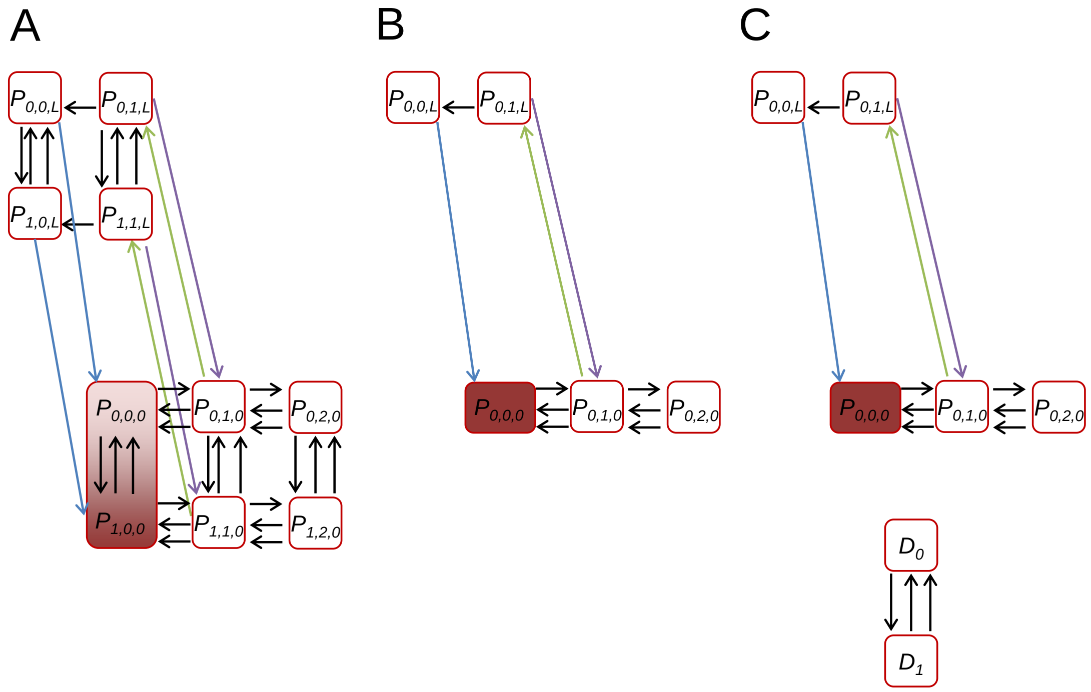

Figure S1 - Transition of Protein-binding status in promoters and decoy sites.
Transition of Protein-binding status in a promoter of a reporter gene and a decoy are shown. (A) lac/ara-reporter circuit. (B) the lac-reporter circuit. (C) the lac-reporter + AraC decoy circuit. Note that all of the three circuits also have the same regulatory circuits with AraC-coding and LacI-coding genes both of which have the same promoter with the lac/ara-reporter gene.

Figure S2 – DNA sequences of the plasmids and primers.

DNA sequence of pPlac-*gfp*_partial

gfp N-terminal partial sequence 507 bp (green)
RBS (blue)
lac promoter (red)

CCCGTGTAAAACGACGGCCAGTTTATCTAGTCAGCTTGATTCTAGCTGATCGTGGA
CCGGAAGGTGAGCCAGTGAGTTGATTGCAGTCCAGTTACGCTGGAGTCTGAGGCT
CGTCCTGAATGATATGCGACCGCCGGAGGGTTGCGTTTGAGACGGGCGACAGATC
CAGTCGCGCTGCTCTCGTCGATCCgtgtctaattttgaagttaactttgataccattcttttgtttgtcagcca
tgatgtaaacattgtgagagttatagttgtattccaatttgtgacctaaaatgttaccatcttctttaaaatcaataccttttaat
tcgattctattaactaaggtatcaccttcaaacttgacttcagctctggtcttgtagttaccgtcatctttgaaaaaaatagttct
ttcttgaacataaccttctggcatggcagacttgaaaaagtcatgttgtttcatatgatctgggtatctagaaaaacattgaac
accataagttaaagtagtgactaaggttggccatggaactggcaatttaccagtagtacaaataaattttaaggtcaattta
ccgtaagtagcatcaccttcaccttcaccggagacagaaaatttgtgaccattaacatcaccatctaattcaaccaaaattgg
gacaacaccagtgaataattcttcacctttagacatggtacctttctcctctttaatgaattctgtgtgaaattgttatccgctca
caattgaatctagtatcattgtgaggctcacaattgtcaagcgactcgaacactaatcatatgctatactctttatggctcgagt
cgacagttcataggtgattgctcaggacatttctgttagaaggaatcgttttccttacttttccttacgcacaagagttccgtag
ctgttcaagtttgtgtttcaactgttctcgtcgtttccgcaacaagtcctcttcagaaatgagcttttgctcctctgcttggacgga
caggatgtatgctgtggctttttttaaggataactaccttgggggccttttcattgttttccaactccgggatctggtcacgcagg
gcaaaaaagctccgttttagctcgttcctcctctggcgctccaagacgttgtgtgttcgcctcttgacattctcctcggtgtccgag
ggccctgtgtgaaattgttatccgctcacaattccacacagctagccctagggcggcggatGGTGCGAGCGGATCG
AGCAGTGTCGATCAGTTCTGGACGAGCGAGCTGTCGTCCGACCCGTGATCTTACG
GCATTATACGTATGATCGGTCCACGATCAGCTAGATTATCTAGTCAGCTTGATGTCA
TAGCTGTTTCCTGAGGCTCAATACTGACCATTTAAATCATACCTGACCTCCATAGCA
GAAAGTCAAAAGCCTCCGACCGGAGGCTTTTGACTTGATCGGCACGTAAGAGGTT
CCAACTTTCACCATAATGAAATAAGATCACTACCGGGCGTATTTTTTGAGTTATCGA
GATTTTCAGGAGCTAAGGAAGCTAAAATGAGTATTCAACATTTCCGTGTCGCCCTTA
TTCCCTTTTTTGCGGCATTTTGCCTTCCTGTTTTTGCTCACCCAGAAACGCTGGTGA
AAGTAAAAGATGCTGAAGATCAGTTGGGTGCACGAGTGGGTTACATCGAACTGGAT
CTCAACAGCGGTAAGATCCTTGAGAGTTTACGCCCCGAAGAACGTTTTCCAATGAT
GAGCACTTTTAAAGTTCTGCTATGTGGCGCGGTATTATCCCGTATTGACGCCGGGC
AAGAGCAACTCGGTCGCCGCATACACTATTCTCAGAATGACTTGGTTGAGTACTCA
CCAGTCACAGAAAAGCATCTCACGGATGGCATGACAGTAAGAGAATTATGCAGTGC

TGCCATAACCATGAGTGATAACACTGCGGCCAACTTACTTCTGGCAACGATCGGAG
GACCGAAGGAGCTAACCGCTTTTTTGCACAACATGGGGGATCATGTAACTCGCCTT
GATCGTTGGGAACCGGAGCTGAATGAAGCCATACCAAACGACGAGCGTGACACCA
CGATGCCTGTAGCAATGGCAACAACGTTGCGCAAACTATTAACTGGCGAACTACTT
ACTCTAGCTTCCCGGCAACAATTAATAGACTGGATGGAGGCGGATAAAGTTGCAGG
ATCACTTCTGCGCTCGGCCCTCCCGGCTGGCTGGTTTATTGCTGATAAATCTGGAG
CCGGTGAGCGTGGGTCTCGCGGTATCATTGCAGCACTGGGGCCAGATGGTAAGCC
CTCCCGCATCGTAGTTATCTACACGACGGGGAGTCAGGCAACTATGGATGAACGAA
ATAGACAGATCGCTGAGATAGGTGCCTCACTGATTAAGCATTGGTAATGAGGGCCC
AAATGTAATCACCTGGCTCACCTTCGGGTGGGCCTTTCTTGAGGACCTAAATGTAA
TCACCTGGCTCACCTTCGGGTGGGCCTTTCTGCGTTGCTGGCGTTTTTCCATAGGC
TCCGCCCCCCTGACGAGCATCACAAAAATCGATGCTCAAGTCAGAGGTGGCGAAA
CCCGACAGGACTATAAAGATACCAGGCGTTTCCCCCTGGAAGCTCCCTCGTGCGCT
CTCCTGTTCCGACCCTGCCGCTTACCGGATACCTGTCCGCCTTTCTCCCTTCGGGA
AGCGTGGCGCTTTCTCATAGCTCACGCTGTAGGTATCTCAGTTCGGTGTAGGTCGT
TCGCTCCAAGCTGGGCTGTGTGCACGAACCCCCCGTTCAGCCCGACCGCTGCGCC
TTATCCGGTAACTATCGTCTTGAGTCCAACCCGGTAAGACACGACTTATCGCCACT
GGCAGCAGCCACTGGTAACAGGATTAGCAGAGCGAGGTATGTAGGCGGTGCTACA
GAGTTCTTGAAGTGGTGGCCTAACTACGGCTACACTAGAAGAACAGTATTTGGTAT
CTGCGCTCTGCTGAAGCCAGTTACCTCGGAAAAAGAGTTGGTAGCTCTTGATCCGG
CAAACAAACCACCGCTGGTAGCGGTGGTTTTTTTGTTTGCAAGCAGCAGATTACGC
GCAGAAAAAAAGGATCTCAAGAAGATCCTTTGATTTTCTACCGAAGAAAGGCCCA

DNA sequence of pMK171

araC constitutive expression region (purple)
gfp (green)
RBS (blue)
lac/ara promoter (red)

AATTCGCGGCCGCTTCTAGAGttatgacaacttgacggctacatcattcactttttcttcacaaccggcacgga
actcgctcgggctggccccggtgcatttttaaatacccgcgagaaatagagttgatcgtcaaaaccaacattgcgaccgacg
gtggcgataggcatccgggtggtgctcaaaagcagcttcgcctggctgatacgttggtcctcgcgccagcttaagacgctaatc
cctaactgctggcggaaaagatgtgacagacgcgacggcgacaagcaaacatgctgtgcgacgctggcgatatcaaaattg
ctgtctgccaggtgatcgctgatgtactgacaagcctcgcgtacccgattatccatcggtggatggagcgactcgttaatcgctt

ccatgcgccgcagtaacaattgctcaagcagatttatcgccagcagctccgaatagcgcccttccccttgcccggcgttaatga
tttgcccaaacaggtcgctgaaatgcggctggtgcgcttcatccgggcgaaagaaccccgtattggcaaatattgacggccag
ttaagccattcatgccagtaggcgcgcggacgaaagtaaacccactggtgataccattcgcgagcctccggatgacgaccgt
agtgatgaatctctcctggcgggaacagcaaaatatcacccggtcggcaaacaaattctcgtccctgatttttcaccacccct
gaccgcgaatggtgagattgagaatataacctttcattcccagcggtcggtcgataaaaaaatcgagataaccgttggcctc
aatcggcgttaaacccgccaccagatgggcattaaacgagtatcccggcagcaggggatcattttgcgcttcagccatactttt
catactcactagtagcggccgccatgttctttcctgcgttatcccctgattctgtggataaccgtattaccgcctttgagtgagct
gataccgctcgccgcagccgaacgccctaggtctagggcggcggatttgtcctactcaggagagcgttcaccgacaaacaac
agataaaacgaaaggcccagtctttcgactgagcctttcgttttatttgatgcctctagcacgcgtctagatcagctaattaag
ctttcacgctgcaagggcgtaattttcgtcgttcgctgcactagttttgtacaattcatccataccatgggtaataccagcagca
gtaacaaattctaacaagaccatgtggtctctcttttcgtttggatctttggataatttagattgagtggataagtaatggttgt
ctggtaacaagactggaccatcaccaattggagtattttgttgataatggtcagctaattgaacagaaccatcttcaatgttgt
gtctaattttgaagttaactttgataccattcttttgtttgtcagccatgatgtaaacattgtgagagttatagttgtattccaat
ttgtgacctaaaatgttaccatcttctttaaaatcaataccttttaattcgattctattaactaaggtatcaccttcaaacttgac
ttcagctctggtcttgtagttaccgtcatctttgaaaaaaatagttctttcttgaacataaccttctggcatggcagacttgaaa
aagtcatgttgtttcatatgatctgggtatctagaaaaacattgaacaccataagttaaagtagtgactaaggttggccatgg
aactggcaatttaccagtagtacaaataaattttaaggtcaatttaccgtaagtagcatcaccttcaccttcaccggagacag
aaaatttgtgaccattaacatcaccatctaattcaaccaaaattgggacaacaccagtgaataattcttcacctttagacatg
gtacctttctcctctttaatgaattctgtgtgaaattgttatccgctcacaattgaatctagtatcattgtgaggctcacaattgtc
aagcgactcgaacactaatcatatgctatactctttatggctcgagtcgacagttcataggtgattgctcaggacatttctgtta
gaaggaatcgttttccttacttttccttacgcacaagagttccgtagctgttcaagtttgtgtttcaactgttctcgtcgtttccgc
aacaagtcctcttcagaaatgagcttttgctcctctgcttggacggacaggatgtatgctgtggctttttttaaggataactacct
tgggggccttttcattgttttccaactccgggatctggtcacgcagggcaaaaaagctccgttttagctcgttcctcctctggcgc
tccaagacgttgtgtgttcgcctcttgacattctcctcggtgtccgagggccctgtgtgaaattgttatccgctcacaattccaca
caCCTAGGTCTAGGTCCGGCAAAAAAACGGGCAAGGTGTCACCACCCTGCCCTTTT
TCTTTAAAACCGAAAAGATTACTTCGCGTTATGCAGGCTTCCTCGCTCACTGACTC
GCTGCGCTCGGTCGTTCGGCTGCGGCGAGCGGTATCAGCTCACTCAAAGGCGGTA
ATCTCGAGTCCCGTCAAGTCAGCGTAATGCTCTGCCAGTGTTACAACCAATTAACC
AATTCTGATTAGAAAAACTCATCGAGCATCAAATGAAACTGCAATTTATTCATATCA
GGATTATCAATACCATATTTTTGAAAAAGCCGTTTCTGTAATGAAGGAGAAAACTCA
CCGAGGCAGTTCCATAGGATGGCAAGATCCTGGTATCGGTCTGCGATTCCGACTCG
TCCAACATCAATACAACCTATTAATTTCCCCTCGTCAAAAATAAGGTTATCAAGTGA
GAAATCACCATGAGTGACGACTGAATCCGGTGAGAATGGCAAAAGCTTATGCATTT
CTTTCCAGACTTGTTCAACAGGCCAGCCATTACGCTCGTCATCAAAATCACTCGCA
TCAACCAAACCGTTATTCATTCGTGATTGCGCCTGAGCGAGACGAAATACGCGATC
GCTGTTAAAAGGACAATTACAAACAGGAATCGAATGCAACCGGCGCAGGAACACT

GCCAGCGCATCAACAATATTTTCACCTGAATCAGGATATTCTTCTAATACCTGGAAT
GCTGTTTTCCCGGGGATCGCAGTGGTGAGTAACCATGCATCATCAGGAGTACGGAT
AAAATGCTTGATGGTCGGAAGAGGCATAAATTCCGTCAGCCAGTTTAGTCTGACCA
TCTCATCTGTAACATCATTGGCAACGCTACCTTTGCCATGTTTCAGAAACAACTCTG
GCGCATCGGGCTTCCCATACAATCGATAGATTGTCGCACCTGATTGCCCGACATTAT
CGCGAGCCCATTTATACCCATATAAATCAGCATCCATGTTGGAATTTAATCGCGGCC
TCGAGCAAGACGTTTCCCGTTGAATATGGCTCATAACACCCCTTGTATTACTGTTTA
TGTAAGCAGACAGTTTTATTGTTCATGATGATATATTTTTATCTTGTGCAATGTAACA
TCAGAGATTTTGAGACACAACGTGGCTTTGTTGAATAAATCGAACTTTTGCTGAGT
TGAAGGATCAGATCACGCATCTTCCCGACAACGCAGACCGTTCCGTGGCAAAGCA
AAAGTTCAAAATCACCAACTGGTCCACCTACAACAAAGCTCTCATCAACCGTGGCT
CCCTCACTTTCTGGCTGGATGATGGGGCGATTCAGGCCTGGTATGAGTCAGCAACA
CCTTCTTCACGAGGCAGACCTCAGCGCTAGCGGAGTGTATACTGGCTTACTATGTT
GGCACTGATGAGGGTGTCAGTGAAGTGCTTCATGTGGCAGGAGAAAAAAGGCTGC
ACCGGTGCGTCAGCAGAATATGTGATACAGGATATATTCCGCTTCCTCGCTCACTG
ACTCGCTACGCTCGGTCGTTCGACTGCGGCGAGCGGAAATGGCTTACGAACGGGG
CGGAGATTTCCTGGAAGATGCCAGGAAGATACTTAACAGGGAAGTGAGAGGGCCG
CGGCAAAGCCGTTTTTCCATAGGCTCCGCCCCCCTGACAAGCATCACGAAATCTGA
CGCTCAAATCAGTGGTGGCGAAACCCGACAGGACTATAAAGATACCAGGCGTTTCC
CCTGGCGGCTCCCTCGTGCGCTCTCCTGTTCCTGCCTTTCGGTTTACCGGTGTCAT
TCCGCTGTTATGGCCGCGTTTGTCTCATTCCACGCCTGACACTCAGTTCCGGGTAG
GCAGTTCGCTCCAAGCTGGACTGTATGCACGAACCCCCCGTTCAGTCCGACCGCT
GCGCCTTATCCGGTAACTATCGTCTTGAGTCCAACCCGGAAAGACATGCAAAAGCA
CCACTGGCAGCAGCCACTGGTAATTGATTTAGAGGAGTTAGTCTTGAAGTCATGCG
CCGGTTAAGGCTAAACTGAAAGGACAAGTTTTGGTGACTGCGCTCCTCCAAGCCA
GTTACCTCGGTTCAAAGAGTTGGTAGCTCAGAGAACCTTCGAAAAACCGCCCTGCA
AGGCGGTTTTTTCGTTTTCAGAGCAAGAGATTACGCGCAGACCAAAACGATCTCAA
GAAGATCATCTTATTAAGGGGTCTGACGCTCAGTGGAACGAAAACTCACGTTAAGG
GATTTTGGTCATGAGATTATCAAAAAGGATCTTCACCTAGATCCTTTTAAATTAAAA
ATGAAGTTTTAAATCAATCTAAAGTATATATGAGTAAACTTGGTCTGACAGTTACCA
ATGCTTAATCAGTGAGGCACCTATCTCAGCGATCTGTCTATTTCGTTCATCCATAGT
TGCCTGACTCCCCGTCGTGTAGATAACTACGATACGGGAGGGCTTACCATCTGGCC
CCAGTGCTGCAATGATACCGCGAGACCCACGCTCACCGGCTCCAGATTTATCAGCA
ATAAACCAGCCAGCCGGAAGGGCCGAGCGCAGAAGTGGTCCTGCAACTTTATCCG
CCTCCATCCAGTCTATTCCATGGTGCCACCTGACGTCTAAGAAACCATTATTATCAT
GACATTAACCTATAAAAATAGGCGTATCACGAGGCAGAATTTCAGATAAAAAAAATC

CTTAGCTTTCGCTAAGGATGATTTCTGG

DNA sequence of pMK271

araC constitutive expression region (purple)
gfp (green)
RBS (blue)
lac promoter (red)

aattcgcggccgcttctagagttatgacaacttgacggctacatcattcactttttcttcacaaccggcacggaactcgctcggg
ctggccccggtgcatttttaaatacccgcgagaaatagagttgatcgtcaaaaccaacattgcgaccgacggtggcgatagg
catccgggtggtgctcaaaagcagcttcgcctggctgatacgttggtcctcgcgccagcttaagacgctaatccctaactgctg
gcggaaaagatgtgacagacgcgacggcgacaagcaaacatgctgtgcgacgctggcgatatcaaaattgctgtctgccag
gtgatcgctgatgtactgacaagcctcgcgtacccgattatccatcggtggatggagcgactcgttaatcgcttccatgcgccg
cagtaacaattgctcaagcagatttatcgccagcagctccgaatagcgcccttccccttgcccggcgttaatgatttgcccaaac
aggtcgctgaaatgcggctggtgcgcttcatccgggcgaaagaaccccgtattggcaaatattgacggccagttaagccattc
atgccagtaggcgcgcggacgaaagtaaacccactggtgataccattcgcgagcctccggatgacgaccgtagtgatgaatc
tctcctggcgggaacagcaaaatatcacccggtcggcaaacaaattctcgtccctgatttttcaccaccccctgaccgcgaatg
gtgagattgagaatataacctttcattcccagcggtcggtcgataaaaaaatcgagataaccgttggcctcaatcggcgttaa
acccgccaccagatgggcattaaacgagtatcccggcagcaggggatcattttgcgcttcagccatacttttcatactcactag
tagcggccgccatgttctttcctgcgttatcccctgattctgtggataaccgtattaccgcctttgagtgagctgataccgctcgcc
gcagccgaacgccctaggtctagggcggcggatttgtcctactcaggagagcgttcaccgacaaacaacagataaaacgaa
aggcccagtctttcgactgagcctttcgttttatttgatgcctctagcacgcgtctagatcagctaattaagctttcacgctgcaa
gggcgtaattttcgtcgttcgctgcactagttttgtacaattcatccataccatgggtaataccagcagcagtaacaaattctaa
caagaccatgtggtctctcttttcgtttggatctttggataatttagattgagtggataagtaatggttgtctggtaacaagact
ggaccatcaccaattggagtattttgttgataatggtcagctaattgaacagaaccatcttcaatgttgtgtctaattttgaagt
taactttgataccattcttttgtttgtcagccatgatgtaaacattgtgagagttatagttgtattccaatttgtgacctaaaatg
ttaccatcttctttaaaatcaataccttttaattcgattctattaactaaggtatcaccttcaaacttgacttcagctctggtcttg
tagttaccgtcatctttgaaaaaaatagttctttcttgaacataaccttctggcatggcagacttgaaaaagtcatgttgtttca
tatgatctgggtatctagaaaaacattgaacaccataagttaaagtagtgactaaggttggccatggaactggcaatttacc
agtagtacaaataaattttaaggtcaatttaccgtaagtagcatcaccttcaccttcaccggagacagaaaatttgtgaccat
taacatcaccatctaattcaaccaaaattgggacaacaccagtgaataattcttcacctttagacatggtacctttctcctcttt
aatgaattctgtgtgaaattgttatccgctcacaattgaatctagtatcattgtgaggctcacaattgtcaagcgactcgaaca
ctaatcatatgctatactctttatggctcgagtcgacagttcataggtgattgctcaggacatttctgttagaaggaatcgtttt
ccttacttttccttacgcacaagagttccgtagctgttcaagtttgtgtttcaactgttctcgtcgtttccgcaacaagtcctcttca
gaaatgagcttttgctcctctgcttggacggacaggatgtatgctgtggctttttaaggataactaccttgggggccttttcatt

gttttccaactccgggatctggtcacgcagggcaaaaaagctccgttttagctcgttcctcctctggcgctccaagacgttgtgt
gttcgcctcttgacattctcctcggtgtccgagggccctgtgtgaaattgttatccgctcacaattccacacacctaggtctaggt
ccggcaaaaaaacgggcaaggtgtcaccaccctgcccttttctttaaaaccgaaaagattacttcgcgttatgcaggcttcct
cgctcactgactcgctgcgctcggtcgttcggctgcggcgagcggtatcagctcactcaaaggcggtaatctcgagtcccgtca
agtcagcgtaatgctctgccagtgttacaaccaattaaccaattctgattagaaaaactcatcgagcatcaaatgaaactgca
atttattcatatcaggattatcaataccatatttttgaaaaagccgtttctgtaatgaaggagaaaactcaccgaggcagttcc
ataggatggcaagatcctggtatcggtctgcgattccgactcgtccaacatcaatacaacctattaatttcccctcgtcaaaaa
taaggttatcaagtgagaaatcaccatgagtgacgactgaatccggtgagaatggcaaaagcttatgcatttctttccagact
tgttcaacaggccagccattacgctcgtcatcaaaatcactcgcatcaaccaaaccgttattcattcgtgattgcgcctgagcg
agacgaaatacgcgatcgctgttaaaaggacaattacaaacaggaatcgaatgcaaccggcgcaggaacactgccagcgc
atcaacaatattttcacctgaatcaggatattcttctaatacctggaatgctgttttcccggggatcgcagtggtgagtaaccat
gcatcatcaggagtacggataaaatgcttgatggtcggaagaggcataaattccgtcagccagtttagtctgaccatctcatc
tgtaacatcattggcaacgctacctttgccatgtttcagaaacaactctggcgcatcgggcttcccatacaatcgatagattgtc
gcacctgattgcccgacattatcgcgagcccatttatacccatataaatcagcatccatgttggaatttaatcgcggcctcgagc
aagacgtttcccgttgaatatggctcataacaccccttgtattactgtttatgtaagcagacagttttattgttcatgatgatat
atttttatcttgtgcaatgtaacatcagagattttgagacacaacgtggctttgttgaataaatcgaacttttgctgagttgaa
ggatcagatcacgcatcttcccgacaacgcagaccgttccgtggcaaagcaaaagttcaaaatcaccaactggtccacctac
aacaaagctctcatcaaccgtggctccctcactttctggctggatgatggggcgattcaggcctggtatgagtcagcaacacct
tcttcacgaggcagacctcagcgctagcggagtgtatactggcttactatgttggcactgatgagggtgtcagtgaagtgcttc
atgtggcaggagaaaaaaggctgcaccggtgcgtcagcagaatatgtgatacaggatatattccgcttcctcgctcactgact
cgctacgctcggtcgttcgactgcggcgagcggaaatggcttacgaacggggcggagatttcctggaagatgccaggaagat
acttaacagggaagtgagagggccgcggcaaagccgtttttccataggctccgcccccctgacaagcatcacgaaatctgac
gctcaaatcagtggtggcgaaacccgacaggactataaagataccaggcgtttccccctggcggctccctcgtgcgctctcctgt
tcctgcctttcggtttaccggtgtcattccgctgttatggccgcgtttgtctcattccacgcctgacactcagttccgggtaggcag
ttcgctccaagctggactgtatgcacgaaccccccgttcagtccgaccgctgcgccttatccggtaactatcgtcttgagtccaa
cccggaaagacatgcaaaagcaccactggcagcagccactggtaattgatttagaggagttagtcttgaagtcatgcgccgg
ttaaggctaaactgaaaggacaagttttggtgactgcgctcctccaagccagttacctcggttcaaagagttggtagctcaga
gaaccttcgaaaaaccgccctgcaaggcggttttttcgttttcagagcaagagattacgcgcagaccaaaacgatctcaaga
agatcatcttattaaggggtctgacgctcagtggaacgaaaactcacgttaagggattttggtcatgagattatcaaaaagg
atcttcacctagatccttttaaattaaaaatgaagttttaaatcaatctaaagtatatatgagtaaacttggtctgacagttac
caatgcttaatcagtgaggcacctatctcagcgatctgtctatttcgttcatccatagttgcctgactccccgtcgtgtagataac
tacgatacgggagggcttaccatctggccccagtgctgcaatgataccgcgagacccacgctcaccggctccagatttatcag
caataaaccagccagccggaagggccgagcgcagaagtggtcctgcaactttatccgcctccatccagtctattccatggtgc
cacctgacgtctaagaaaccattattatcatgacattaacctataaaaataggcgtatcacgaggcagaatttcagataaaa
aaaatccttagctttcgctaaggatgatttctgg

First In-Fusion pJS167 forward primer
AAGAATGGTATCAAAGTTAACTTCAAAATTAGACACAAC

First In-Fusion pJS167 reverse primer
GTCCTGAATGATATGGGCCTTTTTACGGTTCCTGGC

First In-Fusion pPlac-gfp_partial forward primer
GTTAACTTTGATACCATTCTTTTGTTTGTCAGCCATG

First In-Fusion pPlac-gfp_partial reverse primer
CATATCATTCAGGACGAGCCTCAGAC

Second In-Fusion forward primer
ACCCTTAGTGACTCCCTAGACCTAGGTGTGTGGAATTG

Second In-Fusion reverse primer
ACTAGTAGCGGCCGCCATGTTCTTTCCTGCGTTATCC

Deletion forward primer
ACTAGTAGCGGCCGCCATGTTCTTTCCTGCGTTATCC

Deletion reverse primer
GAGTATGAAAAGTATGGCTGAAGCG

Third In-Fusion forward primer
AAGGATGATTTCTGGAATTCGCGGCCGCTTCTAGAGT

Third In-Fusion reverse primer
GTTTTTTTGCCGGACCTAGACCTAGGTGTGTGGAATTG

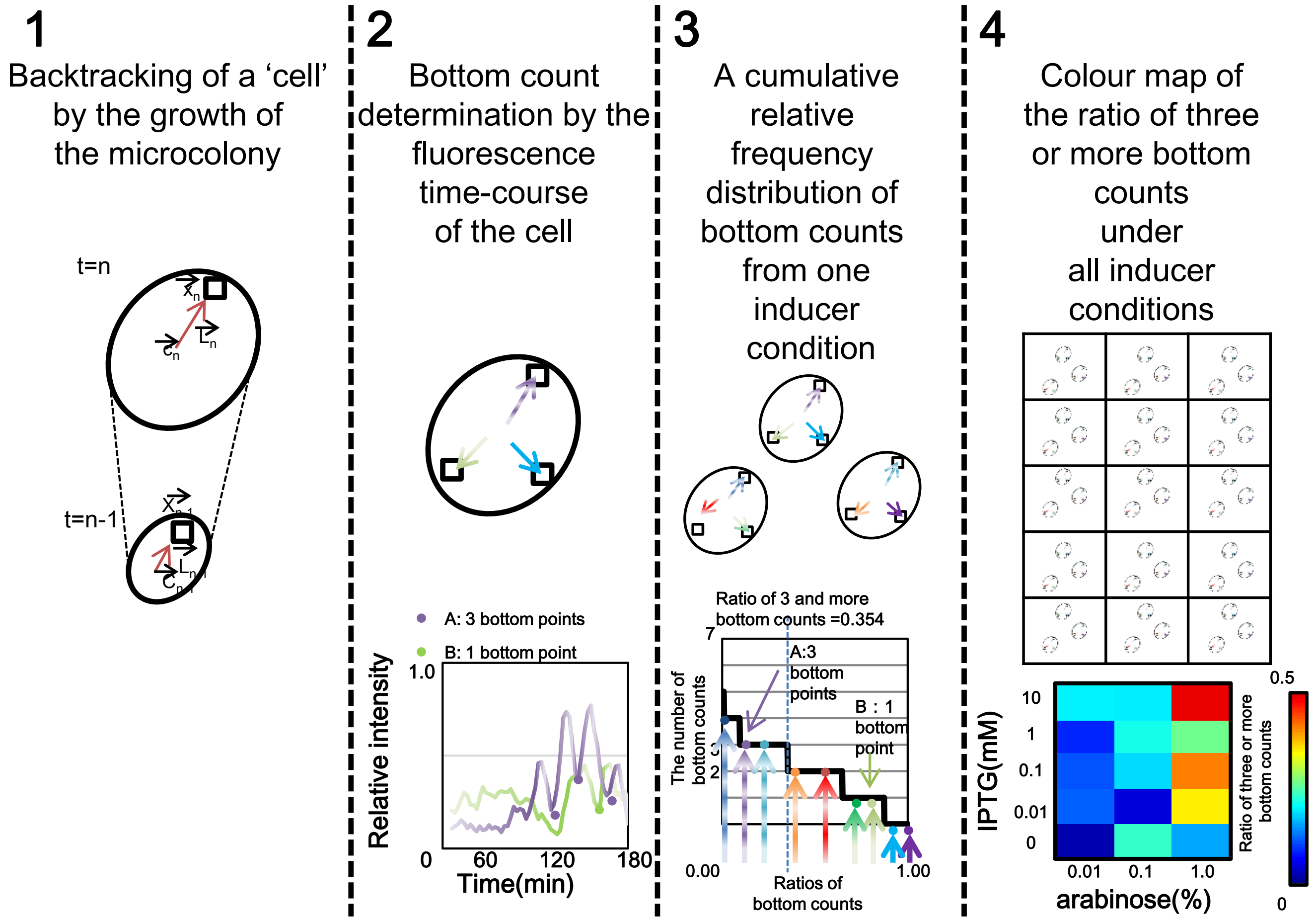

Figure S3 – Imaging process of fluorescence quantification to determine oscillation bottom counts.

To ascertain the strength of the oscillation in noisy conditions, raw image data spread over parameter spaces were collectively evaluated using the following four steps. Fluorescence intensity time-courses were measured for at least four microcolonies from one inducer condition. The detailed process is described in the Supporting Information.

1. Backtracking of a 'cell' by the growth of the microcolony.
2. Bottom count determination by the fluorescence time-course of the cell.
3. Plotting of a cumulative relative frequency distribution of bottom counts from one inducer condition. This distribution for the ratios of bottom counts in descending order (x-axis) and the number of bottom counts (y-axis) is shown.
4. Colour map of the ratio of three or more bottom counts under all inducer conditions from one strain.

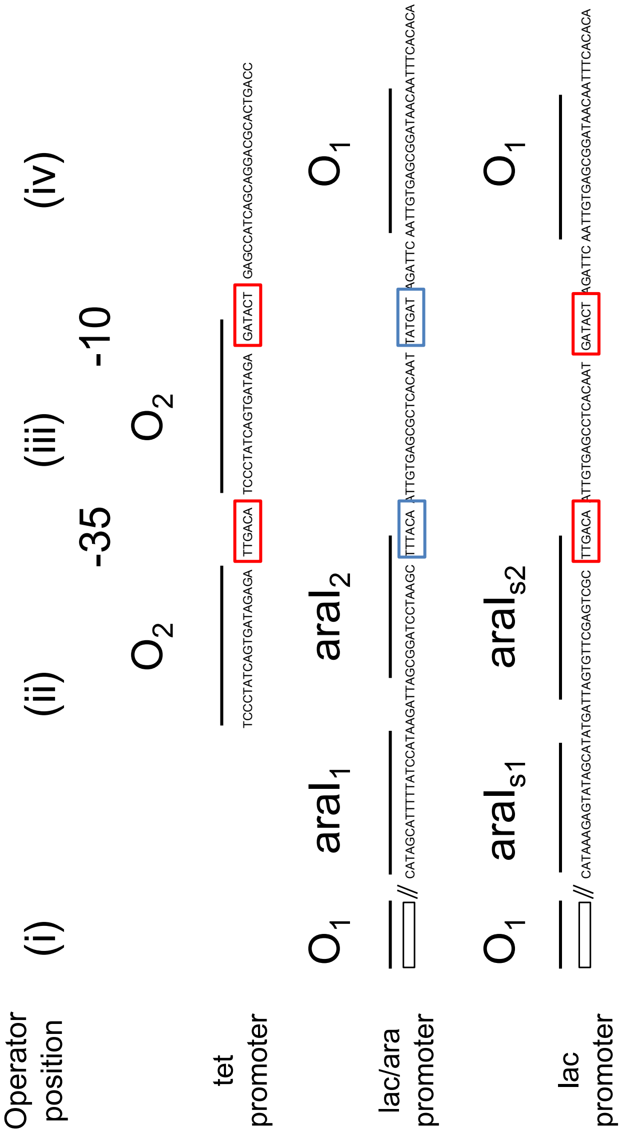

Figure S4 – Composition of the lac promoter.

Schematic diagram of our lac promoter. Red rectangle box indicated strong -35 or -10 sequences. On the other hand, blue rectangle box indicated weak -35 or -10 sequences. O_1 and O_2 are LacI operators that repress the promoter upon binding of LacI protein. $araI_1$ and $araI_2$ are AraC operators that activate the promoter upon binding of AraC protein. $araI_{s1}$ and $araI_{s2}$ are spacer sites that are randomized sequences having the same G/C content with $araI_1$ and $araI_2$ operator sequences, respectively.

GFP intensity of Ptet-*gfp* microscopy experiment

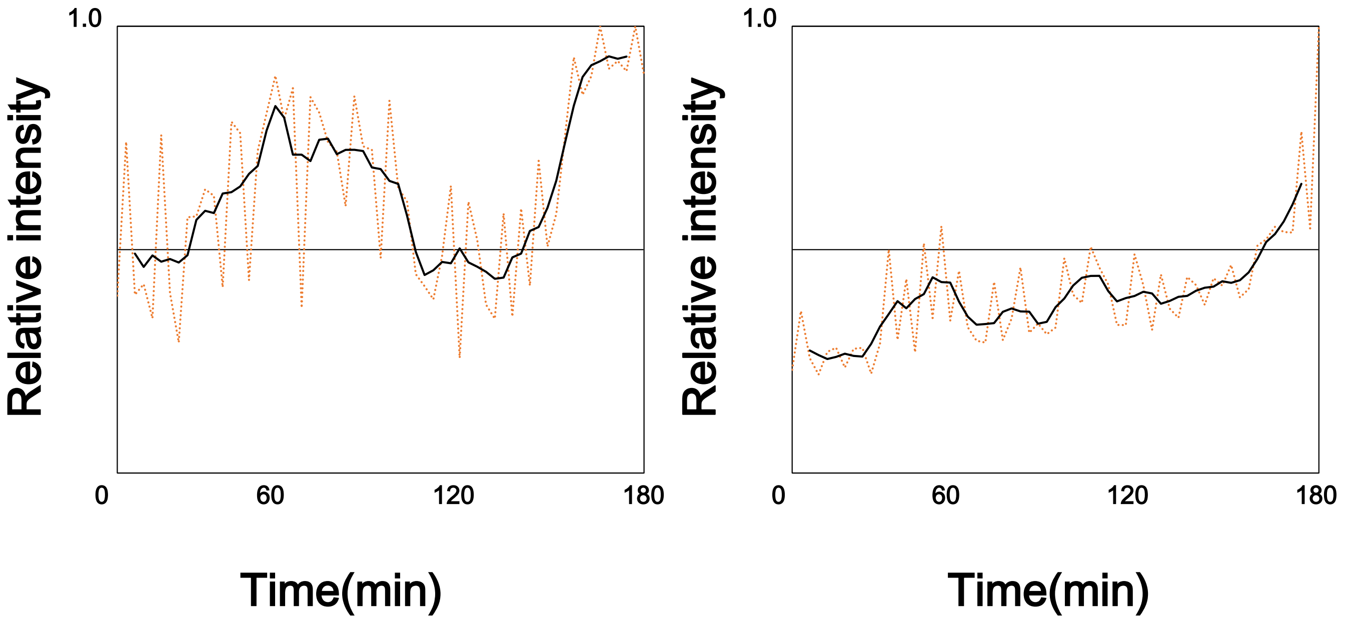

Figure S5 – Fluorescence intensity in the constitutive GFP expressed condition.

Two representative time-courses of GFP fluorescence intensity in the GFP constitutive expressed condition (Ptet-*gfp* strain). The relative fluorescence intensities are shown with red-dashed lines. The 12 per min moving average intensities are shown with black lines.

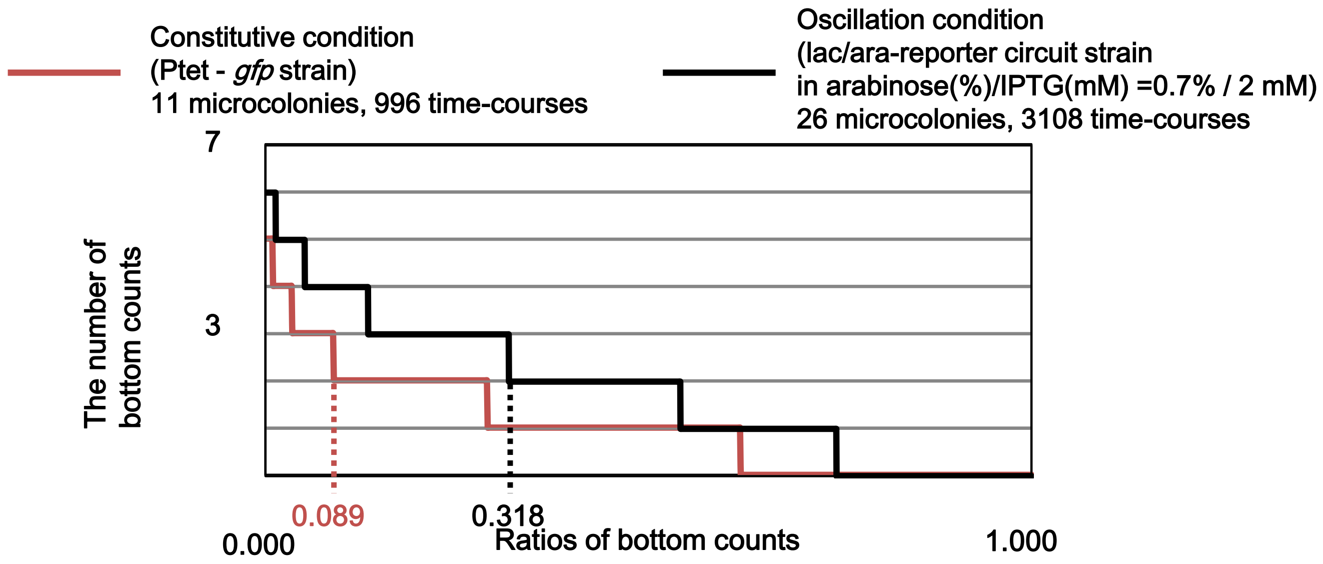

Figure S6. Cumulative relative frequency distributions of the relative bottom counts for the constitutive and oscillation conditions.

The constitutive condition (Ptet-*gfp* strain) is shown with a red line. The oscillation condition (lac/ara-reporter circuit strain in arabinose 0.7% and IPTG 2 mM) is shown with a black line. This distribution is shown with respect to the ratios of bottom counts in descending order from one inducer condition (x-axis), and the number of bottom counts (y-axis). The ratios of three or more bottom counts are shown below the distribution.

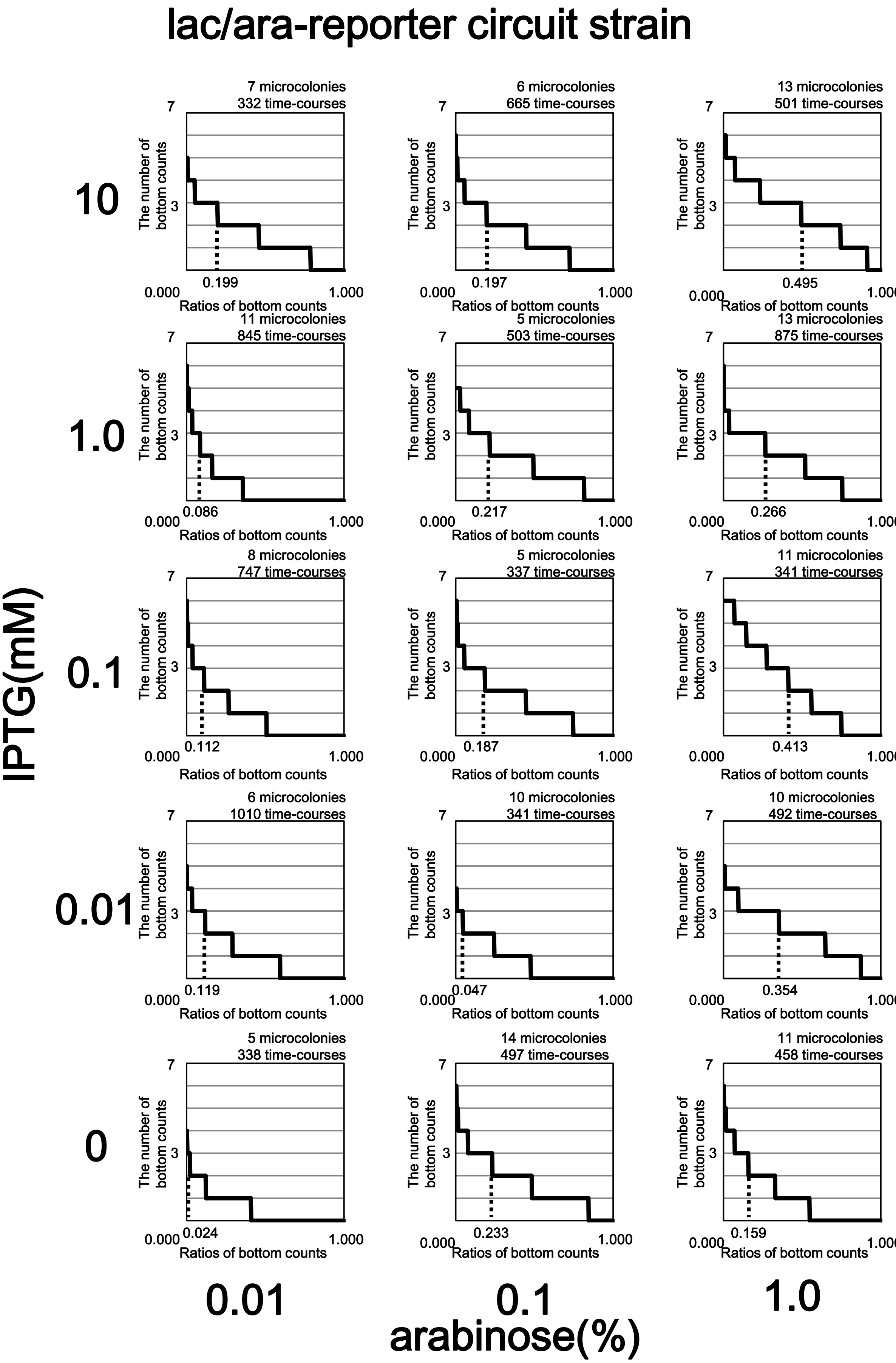

lac/ara-reporter circuit strain
IPTG(mM)
10
1.0
0.1
0.01
0
arabinose(%)
0.01
0.1
1.0
7 microcolonies
332 time-courses
6 microcolonies
665 time-courses
13 microcolonies
501 time-courses
11 microcolonies
845 time-courses
5 microcolonies
503 time-courses
13 microcolonies
875 time-courses
8 microcolonies
747 time-courses
5 microcolonies
337 time-courses
11 microcolonies
341 time-courses
6 microcolonies
1010 time-courses
10 microcolonies
341 time-courses
10 microcolonies
492 time-courses
5 microcolonies
338 time-courses
14 microcolonies
497 time-courses
11 microcolonies
458 time-courses
The number of bottom counts
Ratios of bottom counts
0.199
0.197
0.495
0.086
0.217
0.266
0.112
0.187
0.413
0.119
0.047
0.354
0.024
0.233
0.159

Figure S7. Cumulative relative frequency distributions of bottom counts for the lac/ara-reporter circuit strain.

Cumulative relative frequency distributions of the bottom counts for the lac/ara-reporter circuit strain are plotted for all inducer conditions (arabinose: three points composed of 0.01, 0.1 and 1.0%; IPTG: five points composed of 0.0, 0.01, 0.1, 1.0 and 10.0 mM). These distributions are shown concerning the ratios of bottom counts in descending order from one inducer condition (x-axis), and the number of bottom counts (y-axis). The ratios of three or more bottom counts are shown below each distribution.

lac-reporter circuit strain

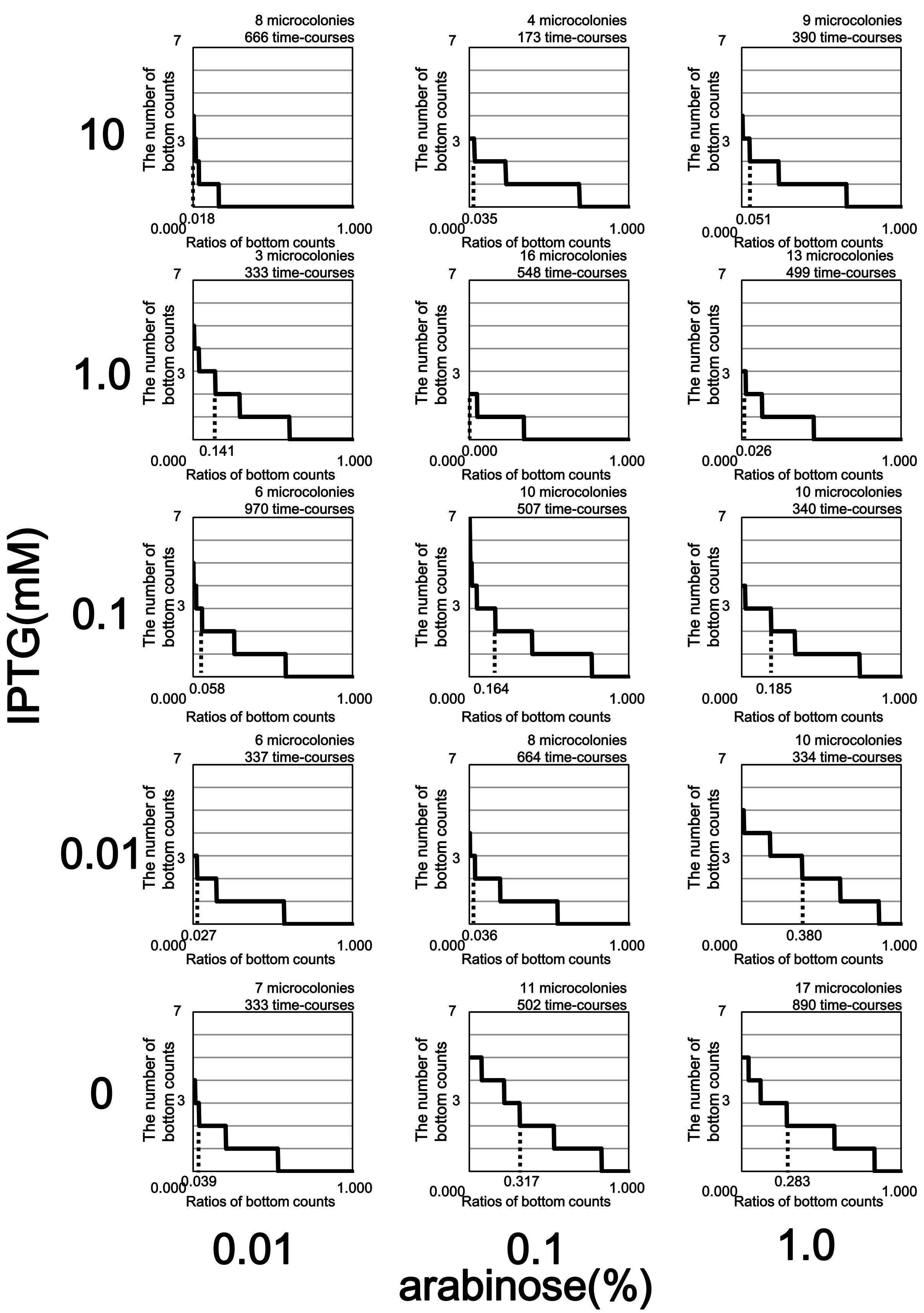

Figure S8 Cumulative relative frequency distributions of bottom counts for the lac-reporter circuit strain.

Cumulative relative frequency distributions of bottom counts for the lac-reporter circuit strain are plotted for all inducer conditions (arabinose: three points composed of 0.01, 0.1 and 1.0%; IPTG: five points composed of 0.0, 0.01, 0.1, 1.0 and 10.0 mM). These distributions are shown concerning the ratios of the bottom counts in descending order from one inducer condition (x-axis), and the number of bottom counts (y-axis). The ratios of three or more bottom counts are shown below each distribution.

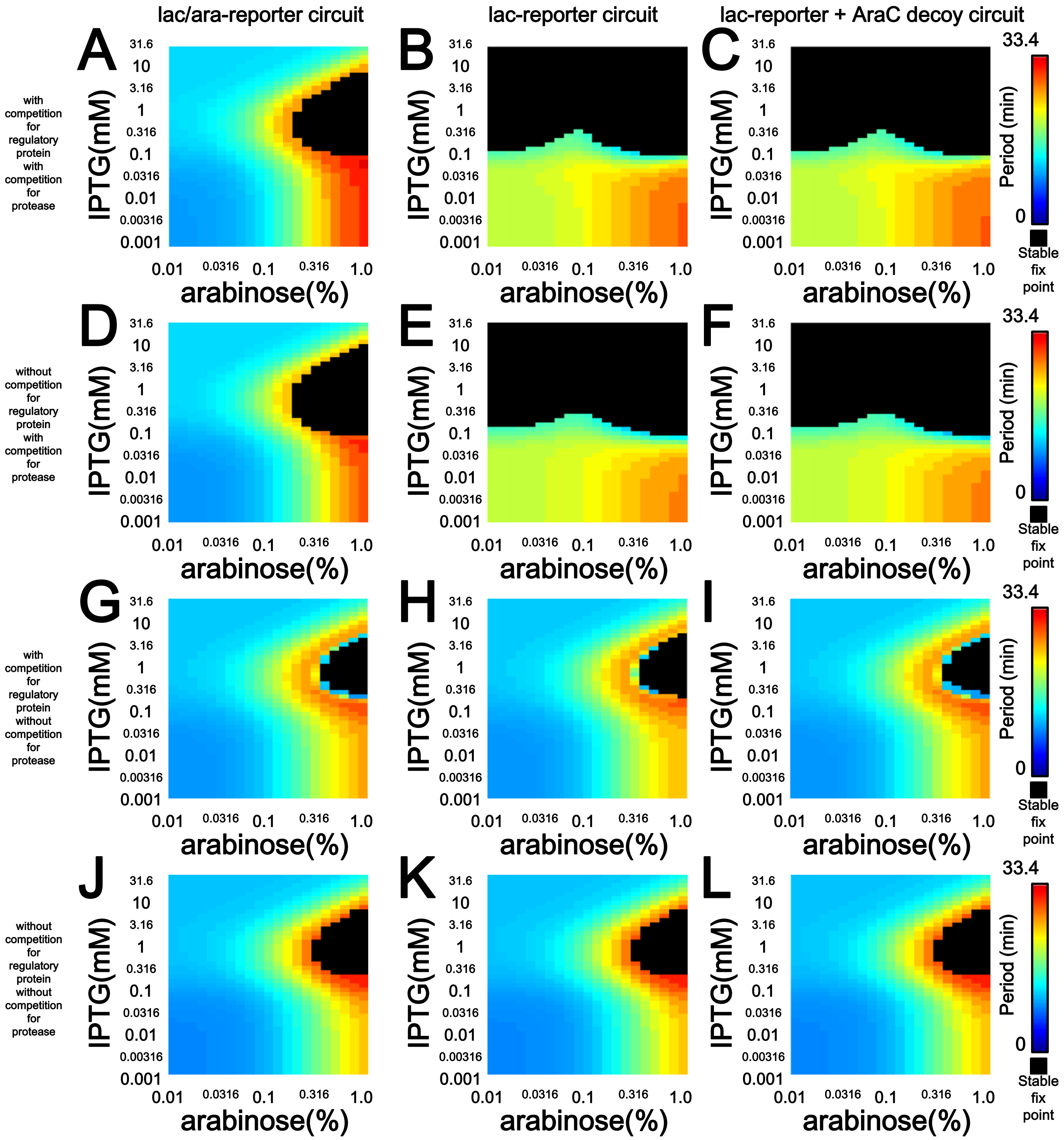

Figure S9. Oscillation periods in the presence or absence of downstream molecular competitions by deterministic simulation.

The behaviors of Smolen oscillators with respect to arabinose (x-axis) and IPTG concentrations (y-axis) are shown by deterministic simulation. The colors in the heat map demonstrate the Oscillation periods. Black regions show stable fixed points.

(A) Oscillation periods produced by the lac/ara-reporter circuit, with downstream competitions from protein-binding sites to their regulatory proteins and from target proteins to their tag-specific proteases.

(B) Oscillation periods produced by the lac-reporter circuit, with downstream competitions from protein-binding sites to their regulatory proteins and from target proteins to their tag-specific proteases.

(C) Oscillation periods produced by the lac-reporter + AraC decoy circuit, with downstream competitions from protein-binding sites to their regulatory proteins and from target proteins to their tag-specific proteases.

(D) Oscillation periods produced by the lac/ara-reporter circuit, without downstream competition from protein-binding sites to their regulatory proteins and with downstream competition from target proteins to their tag-specific proteases.

(E) Oscillation periods produced by the lac-reporter circuit, without downstream competition from protein-binding sites to their regulatory proteins and with downstream competition from target proteins to their tag-specific proteases.

(F) Oscillation periods produced by the lac-reporter + AraC decoy circuit, without downstream competition from protein-binding sites to their regulatory proteins and with downstream competition from target proteins to their tag-specific proteases.

(G) Oscillation periods produced by the lac/ara-reporter circuit, with downstream competition from protein-binding sites to their regulatory proteins and without downstream competition from target proteins to their tag-specific proteases.

(H) Oscillation periods produced by the lac-reporter circuit, with downstream competition from protein-binding sites to their regulatory proteins and without downstream competition from target proteins to their tag-specific proteases.

(I) Oscillation periods produced by the lac-reporter + AraC decoy circuit, with downstream competition from protein-binding sites to their regulatory proteins and without downstream competition from target proteins to their tag-specific proteases.

(J) Oscillation periods produced by the lac/ara-reporter circuit, without downstream competitions from protein-binding sites to their regulatory proteins and from target proteins to their tag-specific proteases.

(K) Oscillation periods produced by the lac-reporter circuit, without downstream competitions from protein-binding sites to their regulatory proteins and from target proteins to their tag-specific proteases.

(L) Oscillation periods produced by the lac-reporter + AraC decoy circuit, without downstream competitions from protein-binding sites to their regulatory proteins and from target proteins to their tag-specific proteases.

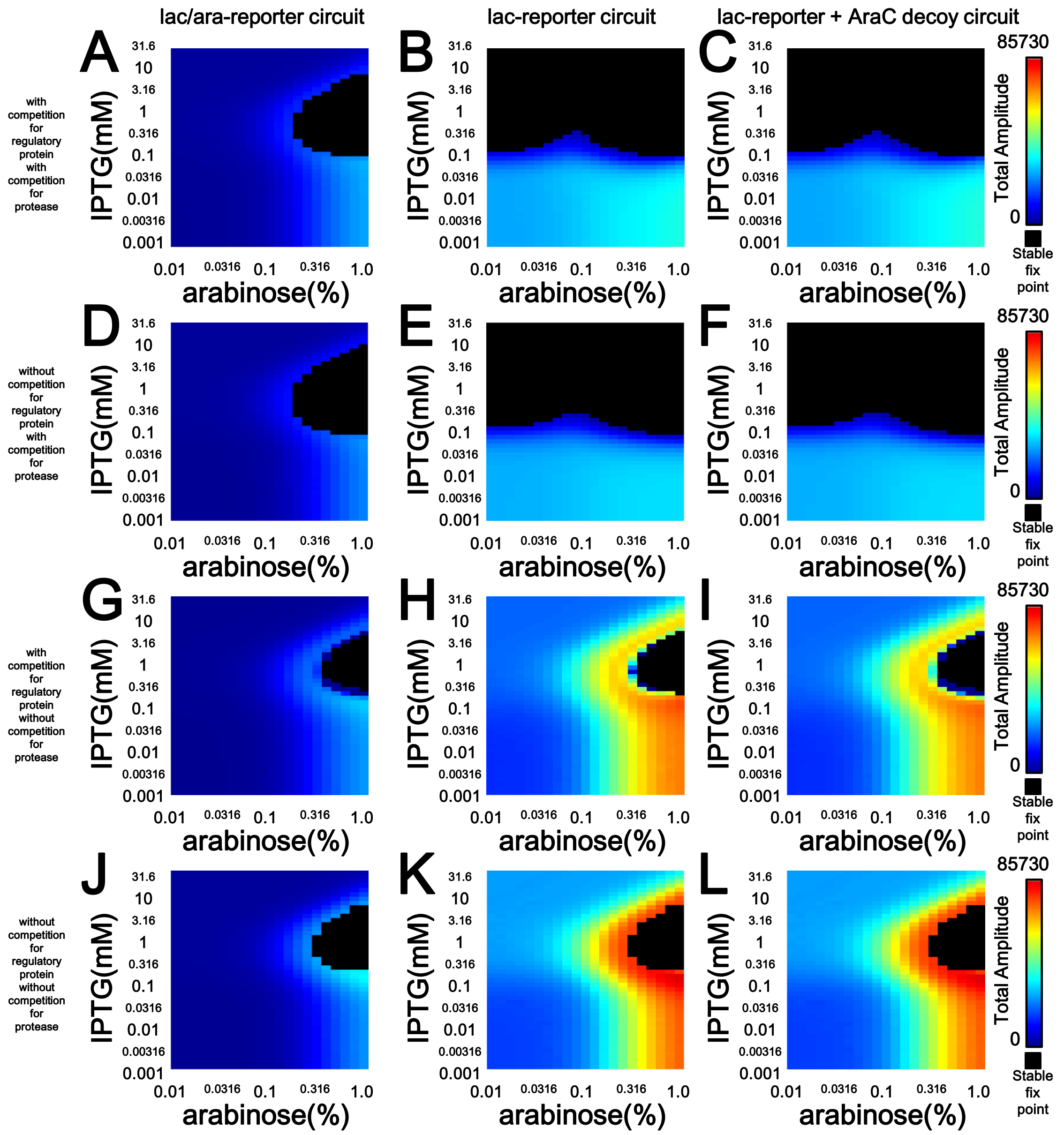

Figure S10. Oscillation Amplitude of total proteins (AraC, LacI, and GFP) in the presence or absence of downstream molecular competitions by deterministic simulation.

The behaviors of Smolen oscillators with respect to arabinose (x-axis) and IPTG concentrations (y-axis) are shown by deterministic simulation. The colors in the heat map demonstrate the amplitude of AraC in the oscillation. Black regions show stable fixed points.

(A) Oscillation amplitude of total proteins produced by the lac/ara-reporter circuit, with downstream competitions from protein-binding sites to their regulatory proteins and from target proteins to their tag-specific proteases.

(B) Oscillation amplitude of total proteins produced by the lac-reporter circuit, with downstream competitions from protein-binding sites to their regulatory proteins and from target proteins to their tag-specific proteases.
(C) Oscillation amplitude of total proteins produced by the lac-reporter + AraC decoy circuit, with downstream competitions from protein-binding sites to their regulatory proteins and from target proteins to their tag-specific proteases.
(D) Oscillation amplitude of total proteins produced by the lac/ara-reporter circuit, without downstream competition from protein-binding sites to their regulatory proteins and with downstream competition from target proteins to their tag-specific proteases.
(E) Oscillation amplitude of total proteins produced by the lac-reporter circuit, without downstream competition from protein-binding sites to their regulatory proteins and with downstream competition from target proteins to their tag-specific proteases.
(F) Oscillation amplitude of total proteins produced by the lac-reporter + AraC decoy circuit, without downstream competition from protein-binding sites to their regulatory proteins and with downstream competition from target proteins to their tag-specific proteases.
(G) Oscillation amplitude of total proteins produced by the lac/ara-reporter circuit, with downstream competition from protein-binding sites to their regulatory proteins and without downstream competition from target proteins to their tag-specific proteases.
(H) Oscillation amplitude of total proteins produced by the lac-reporter circuit, with downstream competition from protein-binding sites to their regulatory proteins and without downstream competition from target proteins to their tag-specific proteases.
(I) Oscillation amplitude of total proteins produced by the lac-reporter + AraC decoy circuit, with downstream competition from protein-binding sites to their regulatory proteins and without downstream competition from target proteins to their tag-specific proteases.
(J) Oscillation amplitude of total proteins produced by the lac/ara-reporter circuit, without downstream competitions from protein-binding sites to their regulatory proteins and from target proteins to their tag-specific proteases.
(K) Oscillation amplitude of total proteins produced by the lac-reporter circuit, without downstream competitions from protein-binding sites to their regulatory proteins and from target proteins to their tag-specific proteases.
(L) Oscillation amplitude of total proteins produced by the lac-reporter + AraC decoy circuit, without downstream competitions from protein-binding sites to their regulatory proteins and from target proteins to their tag-specific proteases.

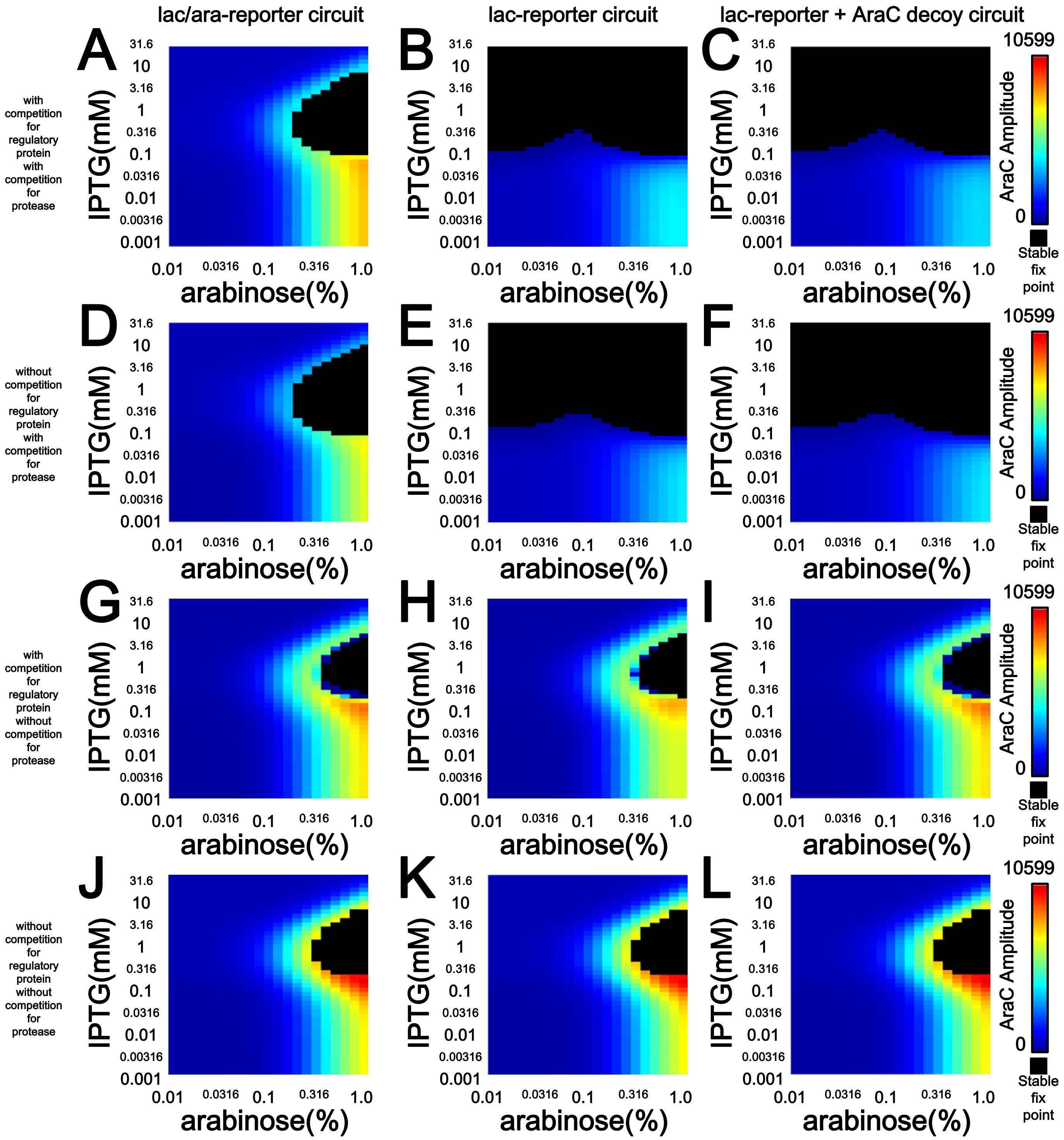

Figure S11. Amplitude of AraC dimer molecule oscillations in the presence or absence of downstream molecular competitions by deterministic simulation.

The behaviors of Smolen oscillators with respect to arabinose (x-axis) and IPTG concentrations (y-axis) are shown by deterministic simulation. The colors in the heat map demonstrate the amplitude of AraC in the oscillation. Black regions show stable fixed points.

(A) AraC amplitude of oscillations produced by the lac/ara-reporter circuit, with downstream competitions from protein-binding sites to their regulatory proteins and from target proteins to their tag-specific proteases.
(B) AraC amplitude of oscillations produced by the lac-reporter circuit, with downstream competitions from protein-binding sites to their regulatory proteins and from target proteins to their tag-specific proteases.
(C) AraC amplitude of oscillations produced by the lac-reporter + AraC decoy circuit, with downstream competitions from protein-binding sites to their regulatory proteins and from target proteins to their tag-specific proteases.
(D) AraC amplitude of oscillations produced by the lac/ara-reporter circuit, without downstream competition from protein-binding sites to their regulatory proteins and with downstream competition from target proteins to their tag-specific proteases.
(E) AraC amplitude of oscillations produced by the lac-reporter circuit, without downstream competition from protein-binding sites to their regulatory proteins and with downstream competition from target proteins to their tag-specific proteases.
(F) AraC amplitude of oscillations produced by the lac-reporter + AraC decoy circuit, without downstream competition from protein-binding sites to their regulatory proteins and with downstream competition from target proteins to their tag-specific proteases.
(G) AraC amplitude of oscillations produced by the lac/ara-reporter circuit, with downstream competition from protein-binding sites to their regulatory proteins and without downstream competition from target proteins to their tag-specific proteases.
(H) AraC amplitude of oscillations produced by the lac-reporter circuit, with downstream competition from protein-binding sites to their regulatory proteins and without downstream competition from target proteins to their tag-specific proteases.
(I) AraC amplitude of oscillations produced by the lac-reporter + AraC decoy circuit, with downstream competition from protein-binding sites to their regulatory proteins and without downstream competition from target proteins to their tag-specific proteases.
(J) AraC amplitude of oscillations produced by the lac/ara-reporter circuit, without downstream competitions from protein-binding sites to their regulatory proteins and from target proteins to their tag-specific proteases.
(K) AraC amplitude of oscillations produced by the lac-reporter circuit, without downstream competitions from protein-binding sites to their regulatory proteins and from target proteins to their tag-specific proteases.
(L) AraC amplitude of oscillations produced by the lac-reporter + AraC decoy circuit, without downstream competitions from protein-binding sites to their regulatory proteins and from target proteins to their tag-specific proteases.

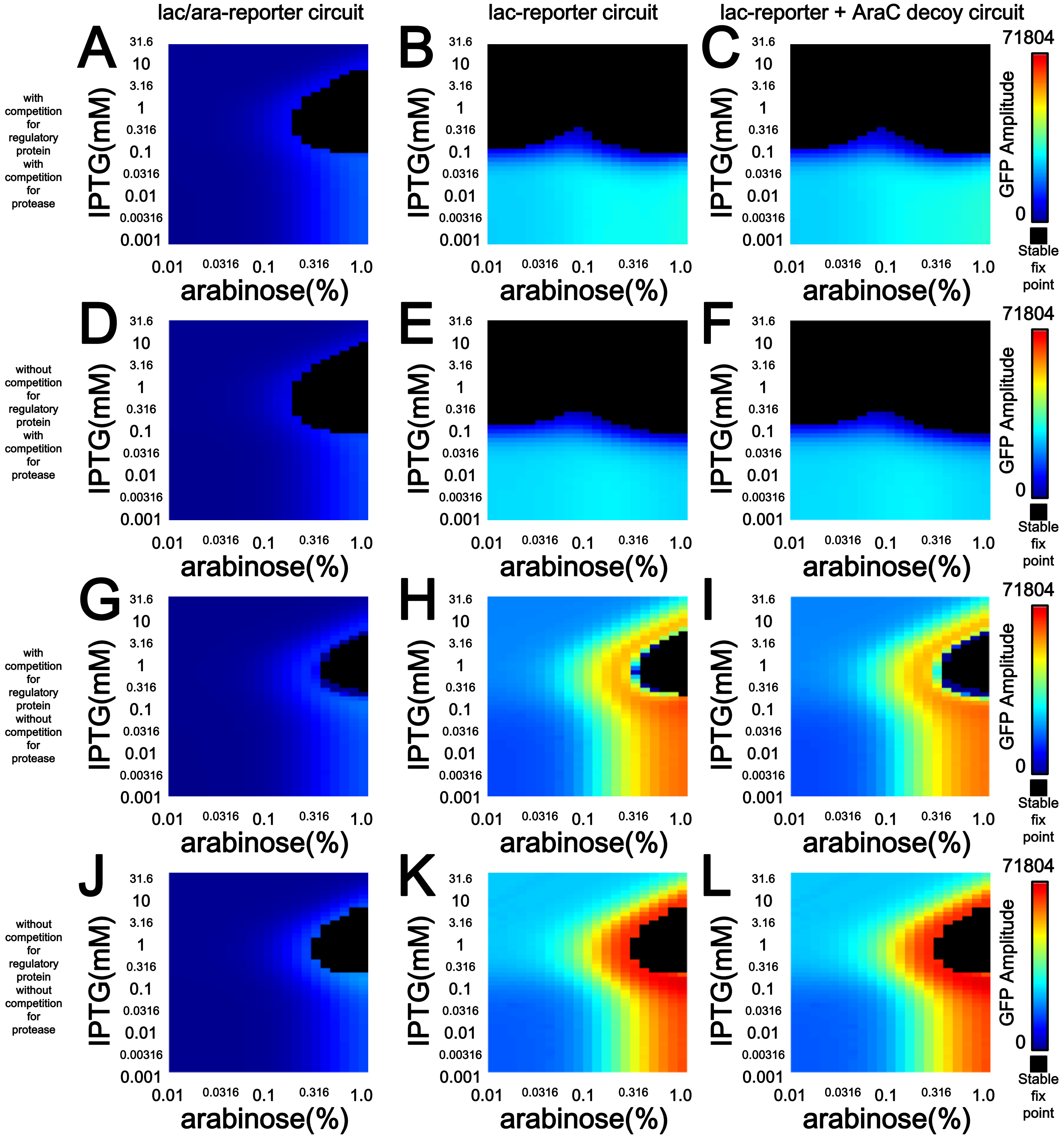

Figure S12. Amplitude of GFP monomer molecule oscillations in the presence or absence of downstream molecular competitions by deterministic simulation.

The behaviors of Smolen oscillators with respect to arabinose (x-axis) and IPTG concentrations (y-axis) are shown by deterministic simulation. The colors in the heat map demonstrate the amplitude of GFP in the oscillation. Black regions show stable fixed points.

(A) GFP amplitude of oscillations produced by the lac/ara-reporter circuit, with downstream competitions from protein-binding sites to their regulatory proteins and from target proteins to their tag-specific proteases.
(B) GFP amplitude of oscillations produced by the lac-reporter circuit, with downstream competitions from protein-binding sites to their regulatory proteins and from target proteins to their tag-specific proteases.
(C) GFP amplitude of oscillations produced by the lac-reporter + AraC decoy circuit, with downstream competitions from protein-binding sites to their regulatory proteins and from target proteins to their tag-specific proteases.
(D) GFP amplitude of oscillations produced by the lac/ara-reporter circuit, without downstream competition from protein-binding sites to their regulatory proteins and with downstream competition from target proteins to their tag-specific proteases.
(E) GFP amplitude of oscillations produced by the lac-reporter circuit, without downstream competition from protein-binding sites to their regulatory proteins and with downstream competition from target proteins to their tag-specific proteases.
(F) GFP amplitude of oscillations produced by the lac-reporter + AraC decoy circuit, without downstream competition from protein-binding sites to their regulatory proteins and with downstream competition from target proteins to their tag-specific proteases.
(G) GFP amplitude of oscillations produced by the lac/ara-reporter circuit, with downstream competition from protein-binding sites to their regulatory proteins and without downstream competition from target proteins to their tag-specific proteases.
(H) GFP amplitude of oscillations produced by the lac-reporter circuit, with downstream competition from protein-binding sites to their regulatory proteins and without downstream competition from target proteins to their tag-specific proteases.
(I) GFP amplitude of oscillations produced by the lac-reporter + AraC decoy circuit, with downstream competition from protein-binding sites to their regulatory proteins and without downstream competition from target proteins to their tag-specific proteases.
(J) GFP amplitude of oscillations produced by the lac/ara-reporter circuit, without downstream competitions from protein-binding sites to their regulatory proteins and from target proteins to their tag-specific proteases.
(K) GFP amplitude of oscillations produced by the lac-reporter circuit, without downstream competitions from protein-binding sites to their regulatory proteins and from target proteins to their tag-specific proteases.
(L) GFP amplitude of oscillations produced by the lac-reporter + AraC decoy circuit, without downstream competitions from protein-binding sites to their regulatory proteins and from target proteins to their tag-specific proteases.

3. Supplementary Movies

Movie SM1. Microscopy image stack movie of the lac/ara-reporter circuit strain at 1.0% arabinose and 10 mM IPTG.

Supplementary movie file 1 showed a time-lapse microscopy movie of the lac/ara-reporter circuit strain continuously induced at 1.0% arabinose and 10 mM IPTG. The DIC image was shown in grey on the left panel, and the GFP fluorescence image was shown in grey on the right panel. Total time of movie was 180 min with a sampling rate of one image every 3 min. Scale bar: 20 μm. The GFP fluorescence image was the original GFP fluorescence image subtracted by background.

Movie SM2. Microscopy image stack movie of the lac-reporter circuit strain at 1.0% arabinose and 10 mM IPTG.

Supplementary movie file 2 showed a time-lapse microscopy movie of the lac-reporter circuit strain continuously induced at 1.0% arabinose and 10 mM IPTG. The DIC image was shown in grey on the left panel, and the GFP fluorescence image was shown in grey on the right panel. Total time of movie was 180 min with a sampling rate of one image every 3 min. Scale bar: 20 μm. The GFP fluorescence image was the original GFP fluorescence image subtracted by background.

Movie SM3. Microscopy image stack movie of the lac/ara-reporter circuit strain at 1.0% arabinose and 0.01 mM IPTG.

Supplementary movie file 3 showed a time-lapse microscopy movie of the lac/ara-reporter circuit strain continuously induced at 1.0% arabinose and 0.01 mM IPTG. The DIC image was shown in grey on the left panel, and the GFP fluorescence image was shown in grey on the right panel. Total time of movie was 180 min with a sampling rate of one image every 3 min. Scale bar: 20 μm. The GFP fluorescence image was the original GFP fluorescence image subtracted by background.

Movie SM4. Microscopy image stack movie of the lac-reporter circuit strain at 1.0% arabinose and 0.01 mM IPTG.

Supplementary movie file 4 showed a time-lapse microscopy movie of the lac-reporter circuit strain continuously induced at 1.0% arabinose and 0.01 mM IPTG. The DIC image was shown in grey on the left panel, and the GFP fluorescence image was shown in grey on the right panel. Total time of movie was 180 min with a sampling rate of one image every 3 min. Scale bar: 20 μm. The GFP fluorescence image was the original GFP fluorescence image subtracted by background.

Movie SM5. Microscopy image stack movie of the GFP constitutive expression (Ptet-*gfp* strain).

Supplementary movie file 5 showed a time-lapse microscopy movie of microscopy image stack movie of the GFP constitutive expression strain (Ptet-*gfp*). The DIC image was shown in grey on the left panel, and the GFP fluorescence image was shown in grey on the right panel. Total time of movie was 180 min with a sampling rate of one image every 3 min. Scale bar: 20 μm. The GFP fluorescence image was the original GFP fluorescence image subtracted by background.
